# Clinical predictors of antipsychotic treatment resistance: Development and internal validation of a prognostic prediction model by the STRATA-G consortium

**DOI:** 10.1016/j.schres.2022.09.009

**Published:** 2022-12

**Authors:** Sophie E. Smart, Deborah Agbedjro, Antonio F. Pardiñas, Olesya Ajnakina, Luis Alameda, Ole A. Andreassen, Thomas R.E. Barnes, Domenico Berardi, Sara Camporesi, Martine Cleusix, Philippe Conus, Benedicto Crespo-Facorro, Giuseppe D'Andrea, Arsime Demjaha, Marta Di Forti, Kim Do, Gillian Doody, Chin B. Eap, Aziz Ferchiou, Lorenzo Guidi, Lina Homman, Raoul Jenni, Eileen Joyce, Laura Kassoumeri, Ornella Lastrina, Ingrid Melle, Craig Morgan, Francis A. O'Neill, Baptiste Pignon, Romeo Restellini, Jean-Romain Richard, Carmen Simonsen, Filip Španiel, Andrei Szöke, Ilaria Tarricone, Andrea Tortelli, Alp Üçok, Javier Vázquez-Bourgon, Robin M. Murray, James T.R. Walters, Daniel Stahl, James H. MacCabe

**Affiliations:** a MRC Centre for Neuropsychiatric Genetics and Genomics, Division of Psychological Medicine and Clinical Neurosciences, Cardiff University, Cardiff, UK; b Department of Psychosis Studies, Institute of Psychiatry, Psychology & Neuroscience, King's College London, London, UK; c Department of Biostatistics & Health Informatics, Institute of Psychiatry, Psychology & Neuroscience, King's College London, London, UK; d Department of Behavioural Science and Health, Institute of Epidemiology and Health Care, University College London, London, UK; e Centro de Investigacion en Red Salud Mental (CIBERSAM), Sevilla, Spain; f Department of Psychiatry, Hospital Universitario Virgen del Rocio, IBiS, Universidad de Sevilla, Spain; g TIPP (Treatment and Early Intervention in Psychosis Program), Service of General Psychiatry, Department of Psychiatry, Lausanne University Hospital (CHUV), Lausanne, Switzerland; h Norwegian Centre for Mental Disorders Research (NORMENT), Institute of Clinical Medicine, University of Oslo, Oslo, Norway; i Division of Mental Health and Addiction, Oslo University Hospital, Oslo, Norway; j Division of Psychiatry, Imperial College London, UK; k Department of Biomedical and Neuro-motor Sciences, Psychiatry Unit, Alma Mater Studiorum Università di Bologna, Bologna, Italy; l Unit for Research in Schizophrenia, Center for Psychiatric Neuroscience, Department of Psychiatry, Lausanne University Hospital (CHUV), Lausanne, Switzerland; m Social Genetics and Developmental Psychiatry, Institute of Psychiatry, Psychology & Neuroscience, King's College London, London, UK; n South London and Maudsley NHS Foundation Trust, London, UK; o Department of Medical Education, University of Nottingham Faculty of Medicine and Health Sciences, Nottingham, UK; p Unit of Pharmacogenetics and Clinical Psychopharmacology, Centre for Psychiatric Neuroscience, Department of Psychiatry, Lausanne University Hospital, University of Lausanne, Prilly, Switzerland; q School of Pharmaceutical Sciences, University of Geneva, University of Lausanne, Geneva, Switzerland; r Center for Research and Innovation in Clinical Pharmaceutical Sciences, University of Lausanne, Switzerland; s Institute of Pharmaceutical Sciences of Western, Switzerland, University of Geneva, University of Lausanne; t Univ Paris Est Creteil, INSERM, IMRB, Translational Neuropsychiatry, Fondation FondaMental, Creteil, France; u AP-HP, Hôpitaux Universitaires H. Mondor, DMU IMPACT, FHU ADAPT, Creteil, France; v Department of Medical and Surgical Sciences, Bologna Transcultural Psychosomatic Team (BoTPT), Alma Mater Studiorum – University of Bologna, Bologna, Italy; w Disability Research Division (FuSa), Department of Behavioural Sciences and Learning, Linköping University, Linköping, Sweden; x Department of Clinical and Movement Neuroscience, UCL Queen Square Institute of Neurology, University College London, London, UK; y Health Service and Population Research, King's College London, London, UK; z Centre for Society and Mental Health, King's College London, London, UK; aa Centre for Public Health, Institute of Clinical Sciences, Queens University Belfast, Belfast, UK; ab Early Intervention in Psychosis Advisory Unit for South East Norway (TIPS Sør-Øst), Division of Mental Health and Addiction, Oslo University Hospital, Norway; ac Department of Applied Neuroscience and Neuroimaging, National Institute of Mental Health, Klecany, Czechia; ad Department of Psychiatry and Medical Psychology, Third Faculty of Medicine, Charles University, Prague, Czechia; ae Groupe Hospitalier Universitaire Psychiatrie Neurosciences Paris, Pôle Psychiatrie Précarité, Paris, France; af Istanbul University, Istanbul Faculty of Medicine, Department of Psychiatry, Istanbul, Turkey; ag Department of Psychiatry, University Hospital Marques de Valdecilla - Instituto de Investigación Marques de Valdecilla (IDIVAL), Santander, Spain; ah Department of Medicine and Psychiatry, School of Medicine, University of Cantabria, Santander, Spain

**Keywords:** Treatment resistant schizophrenia, First episode psychosis, Prospective longitudinal cohort, Prediction modelling, Stratification, Machine learning

## Abstract

**Introduction:**

Our aim was to, firstly, identify characteristics at first-episode of psychosis that are associated with later antipsychotic treatment resistance (TR) and, secondly, to develop a parsimonious prediction model for TR.

**Methods:**

We combined data from ten prospective, first-episode psychosis cohorts from across Europe and categorised patients as TR or non-treatment resistant (NTR) after a mean follow up of 4.18 years (s.d. = 3.20) for secondary data analysis. We identified a list of potential predictors from clinical and demographic data recorded at first-episode. These potential predictors were entered in two models: a multivariable logistic regression to identify which were independently associated with TR and a penalised logistic regression, which performed variable selection, to produce a parsimonious prediction model. This model was internally validated using a 5-fold, 50-repeat cross-validation optimism-correction.

**Results:**

Our sample consisted of N = 2216 participants of which 385 (17 %) developed TR. Younger age of psychosis onset and fewer years in education were independently associated with increased odds of developing TR. The prediction model selected 7 out of 17 variables that, when combined, could quantify the risk of being TR better than chance. These included age of onset, years in education, gender, BMI, relationship status, alcohol use, and positive symptoms. The optimism-corrected area under the curve was 0.59 (accuracy = 64 %, sensitivity = 48 %, and specificity = 76 %).

**Implications:**

Our findings show that treatment resistance can be predicted, at first-episode of psychosis. Pending a model update and external validation, we demonstrate the potential value of prediction models for TR.

## 1 Introduction

Approximately 20–30 % of patients with schizophrenia or a related psychotic disorder continue to experience disabling psychotic symptoms, despite two adequate trials of antipsychotic medication (Mørup et al., 2020; NICE, 2014; Siskind et al., 2022; Stokes et al., 2020). These patients are termed treatment resistant (TR). Patients with TR have a poorer quality of life, poor physical health, and their level of disability often prevents them from working (Brain et al., 2018; Iasevoli et al., 2016; Nordstroem et al., 2017). This, in combination with the cost of medical treatment and hospitalisation, increases the societal economic burden of TR (Andrews et al., 2012; Kennedy et al., 2014). However, many patients with TR can be successfully treated: clozapine is highly effective in reducing symptom severity and mortality rates (Siskind et al., 2017; Warnez and Alessi-Severini, 2014) and there is some evidence that cognitive behavioural therapy (CBT) as an adjunct treatment may be effective (Polese et al., 2019). The problem lies in how long it takes patients to access that treatment. Delays to successful treatment are associated with poorer symptomatic response throughout the course of psychosis (Malla et al., 2006; Murru and Carpiniello, 2018), and a delay in clozapine prescription for patients with TR is associated with poor response to clozapine (Griffiths et al., 2021). One study estimated that clozapine is delayed by an average of four years (Howes et al., 2012). A prediction model capable of identifying TR earlier in the course of illness could help to reduce this delay by identifying patients at higher risk of TR and offering them clozapine earlier in the disease.

Prognostic prediction models are mathematical equations which quantify the predictive power of data at a given time point to predict a future outcome among individuals at risk for that outcome (Hemingway et al., 2013). The equation is optimised to calculate an individual's probability of developing the outcome rather than a group-level association. As a consequence, prediction models can be used to inform clinical decision making (Lee et al., 2016). A single predictor is easy to collect but often has poor predictive power. Conversely, models that require many variables will have better predictive validity but are more cumbersome and difficult to apply across samples with different data; there is thus a trade-off between predictive power and usefulness in real-world settings. Prediction models should therefore include a variable selection step, where the most parsimonious combination of variables is selected. They then undergo model development, internal and external validation, and are in many cases refined, before being implemented in clinical settings. Prognostic prediction models are a useful aid in planning treatment decisions but can also be used to stratify participants by risk in clinical trials (Collins et al., 2015).

Since prediction models require different optimization criteria they thus require different methodologies to classical statistical models (the latter are sometimes referred to as explanatory models) (Hastie et al., 2009; Shmueli, 2010). Machine learning and penalised maximum likelihood methods are often used to develop prediction models (Bzdok and Meyer-Lindenberg, 2018); penalised regression methods are well suited to predicting outcomes with a relatively low prevalence, such as TR (Pavlou et al., 2016).

In a systematic review of predictors of TR, identified in prospective observational cohort studies, only two studies reported statistics that measure predictive validity and all but one used statistical methods designed to identify explanatory variables of TR rather than a prediction model (Smart et al., 2021). Since this review, two further studies have used first-episode cohort data to predict TR. One used a machine learning method, but focused on evaluating individual predictors, not on proposing a model capable of predicting an individual's risk (Legge et al., 2020). The second developed two separate models; one to predict early-onset TR (never experiencing symptomatic remission) and the other to predict late-onset TR (experiencing symptomatic remission before later developing TR) (Ajnakina et al., 2020).

Here we, firstly, report an explanatory model of TR which quantifies the associations between clinical and demographic data collected at first-episode and TR. Secondly, we report the development and internal validation of a prognostic prediction model for TR, using the same set of clinical and demographic variables.

## 2 Methods

We followed guidance set out in the ‘Transparent Reporting of a multivariable prediction model for Individual Prognosis Or Diagnosis’ (TRIPOD) statement to report this study (Collins et al., 2015). The TRIPOD checklist can be found in the Supplementary Material.

### 2.1 Study design and participants

The ‘Schizophrenia: Treatment Resistance and Therapeutic Advances’ (STRATA) Consortium is a UK Medical Research Council-funded, multi-disciplinary group of researchers working together on stratified medicine for schizophrenia and related psychoses. As part of the STRATA-Genetics workstream (STRATA-G), genetic and phenotypic data was collected from existing observational, prospective, first-episode psychosis cohorts to perform secondary data analysis. To be included in STRATA-G, cohorts were required to have data on individuals recruited at first episode and prospectively followed for a minimum of 12 months, and to have included DNA collection in the study protocol. Analysis of genetic data is not reported here, and all participants were included in this analysis regardless of whether their genetic data were available. Details of recruitment, follow up, and where data has been previous published for each cohort can be found in the Supplementary Material. For this study, we removed cohorts where the number of TR cases was less than five (see Supplementary Material). Ten STRATA-G cohorts were included, with data collected in the Czech Republic (Spaniel et al., 2016) (1), France (Jongsma et al., 2018) (1), Italy (Jongsma et al., 2018; Tarricone et al., 2012) (1), Spain (Crespo-Facorro et al., 2007) (1), Switzerland (Baumann et al., 2013) (1), Turkey (Üçok et al., 2011) (1), and the UK (Barnes et al., 2008; Demjaha et al., 2017; Lally et al., 2016; Turkington et al., 2018) (4). All cohorts had ethical approval and participants provided written informed consent. The study was approved by an NHS Research Ethics Committee (15/SC/0021).

### 2.2 Treatment resistance (TR)

Participants were defined as TR if they met any of the following three definitions of TR (criteria varied across cohorts depending on the data available): either (1) prescription of or treatment with clozapine, or (2) treatment with two different antipsychotics at a therapeutic dose for a specified duration (each of at least 6 weeks' duration, with dosages in at least the mid-point of the licensed therapeutic range; data on the reason for switching was not available and therefore not included in the definition of TR), or (3) persistent psychotic symptoms and moderate functional impairment despite treatment with two different antipsychotics at a therapeutic dose for a specified duration of time. Participants not meeting any of these criteria were classified as non-treatment resistant (NTR). Further details on these criteria, broken down by cohort, can be found in Supplementary Table 6.

### 2.3 Predictors

Any variables recorded in more than one cohort, at baseline, were considered as potential predictors. We removed variables where >80 % of the data were missing (Supplementary Table 1), one variable from every pair with a Pearson's correlation >0.8 (Supplementary Table 2) to prevent multicollinearity (retaining the most clinically informative), and categorical variables where the number of events or non-events per category was less than ten.

### 2.4 Statistical analysis

#### 2.4.1 Explanatory model

We imputed missing data using the mice package (van Buuren and Groothuis-Oudshoorn, 2011) for multiple imputation by chained equations (MICE). TR status, length of follow up, and an indicator for cohort were included. We ran MICE using 100 imputations, with ten iterations in the burn-in period. Performance was assessed by plotting the residuals and convergence of chains, exploring the range of imputed values, and comparing observed and imputed data (Nguyen et al., 2017).

We conducted logistic regressions to examine the associations between predictors and TR. Each of the 100 imputed datasets were analysed separately and then the results were pooled together based on Rubin's rules (Azur et al., 2011; Rubin, 1987). Regression coefficients were pooled by taking the average coefficient from all the imputed datasets and standard errors by combining the within imputation variance and the between imputation variance. We report the pooled estimates from univariable analyses and the multivariable model. Length of follow up and cohort were included as covariates in the multivariable model. As a measure of overall model fit, we report the pooled Nagelkerke R^2^ value (Harel, 2009) for the multivariable model.

#### 2.4.2 Prediction model

We imputed missing data using the missForest package (Stekhoven and Buhlmann, 2012) for imputation by random forests (Breiman, 2001). Imputation performance was assessed using the out-of-bag imputation normalized root mean squared error (NRMSE) for continuous imputed data, and the proportion of falsely classified entries (PFC) for the categorical imputed data set (in both cases, a value close to 0 % indicates good performance and a value close to 100 % poor performance; we considered a value around 50 % to indicate a moderate performance) (Stekhoven and Buhlmann, 2012).

We conducted a ‘Least Absolute Shrinkage and regression Operator’ (LASSO) logistic regression to identify a model capable of predicting TR. LASSO is a form of regularised regression which can reduce overfitting when there are a large number of predictors (Hastie et al., 2009). We preferred lasso over other regularization methods (ridge or elastic net) because it tends to select a sparse and, therefore, more clinically practical model (Steyerberg, 2008). The model coefficients are shrunk towards zero, with some coefficients being assigned exactly zero to perform variable selection. The model was fitted using the penalised maximum likelihood procedure implemented in ‘glmnet’. The tuning parameter lambda (λ) was estimated on the imputed data using a grid of 100 tuning parameters. After repeated cross-validation (5 folds, 50 repeats), lambda was selected as the value which maximised the cross-validated area under the receiver operating characteristic curve (AUC) while being within one standard error of the maximum AUC (also known as the “1SE rule”). Simulations suggest that the 1SE model provides the best compromise between reliable variable selection and good prediction accuracy (Hastie et al., 2009). When the number of individuals in one group is substantially larger than the other (in our case, the NTR group), choosing the threshold that maximises accuracy will often produce a model that achieves good overall accuracy by simply classifying all participants into the larger group. Therefore, we chose the ‘best’ threshold, defined as the threshold that maximised the sum of the sensitivity and specificity (Perkins and Schisterman, 2006; Youden, 1950). The apparent performance of the model is presented in the Supplementary Material: we assessed the model performance using calibration (calibration slope and calibration-in-the-large) and discrimination (AUC) and report the model sensitivity (proportion of TR cases correctly predicted), specificity (proportion of NTR cases correctly predicted), positive predictive value (PPV; proportion of predicted TR cases that are indeed TR), negative predictive value (NPV; proportion of predicted NTR cases that are indeed NTR) at the optimal cut-off point for the predicted probability. The optimal cut-off point was defined as the threshold which maximised overall correct classification rates and minimised misclassification rates, while choosing the point on the receiver operating characteristic curve farthest from chance.

In the absence of an independent dataset with which to externally validate the model, we conducted internal, repeated cross-validation (5 folds, 50 repeats) to estimate the optimism of the model (the degree to which the accuracy of the model is inflated by overfitting) and recalibrated the beta coefficients (Steyerberg, 2008). The difference between the training and test performance estimates of each fold were averaged to obtain an estimate of the optimism. The optimism was then subtracted from the apparent performance measures to obtain the corrected (internally validated) performance measures. We report the corrected performance measures and the recalibrated regression coefficients.

#### 2.4.3 Prediction model (LASSO logistic regression) – sensitivity analyses

We refitted the model to examine the stability of the discrimination performance measures across cohorts by using repeated 5-fold cross-validation (5 folds, 50 repeats). In addition, we repeated the model development using (i) a subsample of participants where TR was defined using only clozapine prescription, and (ii) a subsample with a diagnosis of schizophrenia (at the last known follow-up visit). Our main analysis included participants with a range of diagnoses and did not require clozapine treatment as a definition of TR because we wished to maximise our sample size. Our clozapine model uses a subgroup of participants where we are more confident in their classification as TR and our schizophrenia model reflects clinical guidelines which specify a diagnosis of schizophrenia as one of the criteria for TR.

### 2.5 Software

Pre-processing of individual cohorts was conducted in STATA version 14 (Stata Statistical Software: Release 14, StataCorp LP., College Station, TX). Analyses were performed in RStudio (v1.3.1073) using R (v4.0.2) with the exception of MICE and optimism-correction which used R (v4.0.0) in the Hawk cluster of Supercomputing Wales. Scripts used for analysis can be found at: https://github.com/sophiesmart/stratagprediction.

## 3 Results

The final dataset consisted of N = 2216 participants, from ten cohorts, of which 385 (17 %) were TR (Table 1). Data preparation is described in the Supplementary Material, after which we retained 19 predictors. The descriptive statistics for these predictors, as well as length of follow up, clozapine use, and schizophrenia diagnosis, are presented in Table 2. The mean proportion of missing data was 45.68 % (ranging from 0 % to 75.30 %; see Supplementary Table 1).

**Table 1:** STRATA-G cohorts included in this study, stratified by treatment resistant status.

	Non-TR (%)	TR (%)	Total
AESOP London	210 (73.43)	76 (26.57)	286
Belfast	138 (90.20)	15 (9.80)	153
Bologna	43 (86.00)	7 (14.00)	50
GAP London	216 (75.26)	71 (24.74)	287
Istanbul	98 (72.59)	37 (27.41)	135
Lausanne	250 (89.29)	30 (10.71)	280
Paris	26 (81.25)	6 (18.75)	32
Prague	76 (54.29)	64 (45.71)	140
Santander	410 (86.32)	65 (13.68)	475
West London	364 (96.30)	14 (3.70)	378
Total	1831 (82.63)	385 (17.37)	2216

**Table 2:** Summary statistics for the first-episode variables, covariates, and the data used to define subsamples of the data, stratified by treatment resistant status.

	Overall	Non-TR	TR	Statistic^a^	P-value
N	2216	1831	385		
Clozapine (yes; %)	238 (12.00)	0 (0.00)	238 (68.40)		
Length of follow up (years; mean (SD))	4.41 (3.21)	4.17 (2.93)	5.53 (4.12)	6.17	<0.001
Age at onset (years; mean (SD))	26.87 (9.26)	27.35 (9.48)	24.41 (7.56)	−6.02	<0.001
Duration of untreated psychosis (days; mean (SD))	232.41 (619.55)	224.62 (611.71)	276.37 (661.64)	1.20	0.231
Gender (female; %)	820 (39.10)	713 (40.50)	107 (31.70)	9.38	0.002
BMI (mean (SD))	23.87 (4.63)	23.76 (4.71)	24.50 (4.17)	1.52	0.129
Current relationship (yes; %)	300 (19.50)	269 (21.50)	31 (10.90)	16.67	<0.001
Living situation (%)				2.75	0.251
Alone	258 (25.2)	208 (24.9)	50 (26.7)		
With family	667 (65.3)	553 (66.2)	114 (61.0)		
With others	97 (9.5)	74 (8.9)	23 (12.3)		
Employment (employed; %)	213 (39.9)	174 (40.7)	39 (36.4)	0.66	0.461
Education (years; mean (SD))	12.52 (3.17)	12.69 (3.19)	11.73 (2.93)	−2.91	0.004
Cannabis (yes; %)	489 (45.9)	415 (45.6)	74 (48.1)	0.33	0.601
Tobacco (no; %)	423 (43.7)	381 (45.0)	42 (34.7)	4.59	0.043
Alcohol (no; %)	370 (40.5)	339 (42.1)	31 (29.0)	6.71	0.011
PANSS positive subscale (mean (SD))	14.73 (6.04)	14.46 (6.03)	16.00 (5.97)	2.04	0.042
SAPS (mean (SD))	11.39 (3.97)	11.30 (3.95)	12.09 (4.06)	2.02	0.044
SANS (mean (SD))	8.09 (6.51)	7.97 (6.44)	9.07 (7.01)	1.70	0.090
BPRS (mean (SD))	63.02 (13.88)	62.69 (13.79)	64.70 (14.29)	1.32	0.186
GAF (mean (SD))	50.32 (17.07)	51.68 (17.45)	45.46 (14.73)	−3.45	<0.001
Ethnicity (%)				4.67	0.098
European	1071 (70.5)	873 (71.3)	198 (67.1)		
African	308 (20.3)	235 (19.2)	73 (24.7)		
Asian/mixed/other	140 (9.2)	116 (9.5)	24 (8.1)		
Schizophrenia diagnosis (schizophrenia; %)	580 (26.2)	470 (25.7)	110 (28.6)	1.39	0.262

Abbreviations: BMI, Body Mass Index; BPRS, Brief Psychiatric Rating Scale; GAF, Global Assessment of Functioning; PANSS, Positive and Negative Syndrome Scale; SANS, Scale for the Assessment of Negative Symptoms; SAPS, Scale for the Assessment of Positive Symptoms; SD, standard deviation.

a Continuous variables were compared across groups using independent samples *t*-tests and categorical variables were compared across groups using Chi-squared tests (with a Monte Carlo test, with 2000 repeats, to simulate p-values). For continuous variables, a F-test for homogeneity in the variances was performed and, consequently, a Welch Two Sample *t*-test assuming unequal variances was used for length of follow up and age of onset.

### 3.1 Explanatory model

Missing data imputation performed well: chains of trace plots showed convergence, the distributions of the imputed values were consistent with the distributions of the non-imputed values, and the residuals of each imputed dataset showed that the assumptions were met (see Supplementary Material). In univariable logistic regression models, eight predictors were associated with TR. Younger age of onset, being male, higher BMI, not being in a relationship, fewer years in education, higher SAPS score, lower GAF score (worse functioning), and being of African ethnicity (compared to European ethnicity) all increased the odds of being TR (see Supplementary Table 3).

However, in the multivariable logistic regression, controlling for all other predictors as well as cohort and length of follow up, only younger age of onset and fewer years in education increased the odds of being TR (Table 3; Supplementary Table 4). The R^2^ for this model was 26.83 %.

**Table 3:** Results of the multivariable logistic regression (explanatory model).

	Odds ratio	95 % CI		P-value
Age at onset (years)	0.96	0.94	0.98	<0.001***
Duration of untreated psychosis (days)	1.00	1.00	1.00	0.821
Gender (female)	0.89	0.53	1.25	0.518
BMI	1.05	0.99	1.11	0.089
Current relationship (yes)	0.64	0.12	1.17	0.098
Living situation (alone)	0.92	0.43	1.42	0.754
Living situation (with others)	1.52	0.94	2.09	0.160
Employment (employed)	1.03	0.43	1.63	0.922
Education (years)	0.87	0.79	0.95	0.001***
Cannabis (yes)	1.01	0.45	1.57	0.981
Tobacco (no)	1.07	0.35	1.79	0.858
Alcohol (no)	0.87	0.17	1.56	0.686
PANSS positive subscale	1.04	0.95	1.12	0.435
SAPS	1.09	0.98	1.19	0.134
SANS	1.00	0.94	1.07	0.887
BPRS	0.98	0.95	1.02	0.297
GAF	0.98	0.95	1.00	0.059
Ethnicity (African)	1.00	0.15	1.84	0.991
Ethnicity (Asian/Mixed/Other)	0.85	0.19	1.51	0.630

NB: Cohort and length of follow up were included as covariates in the model.

Beta coefficients and standard errors for all predictors and covariates are in Supplementary Table 3 (univariable) and Supplementary Table 4 (multivariable).

The reference category for living situation was ‘living with family’ and for ethnicity the reference category was ‘European’.

Abbreviations: BMI, Body Mass Index; BPRS, Brief Psychiatric Rating Scale; GAF, Global Assessment of Functioning; PANSS, Positive and Negative Syndrome Scale; SANS, Scale for the Assessment of Negative Symptoms; SAPS, Scale for the Assessment of Positive Symptoms.

### 3.2 Prediction model

Missing data imputation performed moderately well: the NRMSE was 52.25 % and PFC was 19.15 %. In the 1SE LASSO regression model, seven variables were selected (Table 4): age of onset, years in education, gender, BMI, relationship status, alcohol use, and positive symptoms. The model predicted TR with an apparent AUC of 0.65 (apparent performance measures for the prediction model and all sensitivity analyses are in the Supplementary Material while the model coefficients are in Supplementary Table 5). The optimal cut-off for the predicted probabilities was 20.21 %. After correcting for optimism, the model AUC was 0.59 (accuracy = 64.33 %, sensitivity = 48.31 %, specificity = 76.19 %, PPV = 27.49 %, and NPV = 87.55 %). When looking across cohorts, the apparent AUC ranged between 0.59 and 0.77 (see Supplementary Material).

**Table 4:** Results of the multivariable LASSO regressions (prediction models).

	Original Model (N = 2216)		Clozapine-only Model (N = 1986)		Schizophrenia-only Model (N = 580)	
	Log Odds	Effect Dir.	Log Odds	Effect Dir.	Log Odds	Effect Dir.
Age at onset (years)	−0.03	−	−0.04	−	−0.02	−
Duration of untreated psychosis (days)						
Gender (female)	−0.09	−			−0.13	−
BMI	0.03	+				
Current relationship (yes)	−0.37	−	−0.06	−	−0.76	−
Living (alone)						
Living (with others)						
Employment (employed)			−0.01	−	−0.35	−
Education (years)	−0.06	−				
Cannabis (yes)						
Tobacco (no)			−0.24	−		
Alcohol (no)	−0.48	−	−0.11	−		
PANSS positive subscale	−0.002	−				
SAPS						
SANS			0.03	+		
BPRS						
GAF						
Ethnicity (African)			0.25	+		
Ethnicity (Asian/Mixed/Other)						

NB: The clozapine-only model was developed using a subsample of participants where TR was defined using only clozapine prescription. The schizophrenia-only model was developed using a subsample of participants with a diagnosis of schizophrenia (at the last known follow-up visit). Apparent and recalibrated regression beta coefficients are presented side by side in Supplementary Table 5.

The reference category for living situation was ‘living with family’ and for ethnicity the reference category was ‘European’.

Abbreviations: BMI, Body Mass Index; BPRS, Brief Psychiatric Rating Scale; GAF, Global Assessment of Functioning; PANSS, Positive and Negative Syndrome Scale; SANS, Scale for the Assessment of Negative Symptoms; SAPS, Scale for the Assessment of Positive Symptoms.

After correcting for optimism, the clozapine-model AUC was 0.61 (accuracy = 55.79 %, sensitivity = 65.97 %, specificity = 55.61 %, PPV = 16.83 %, and NPV = 92.41 %), while the schizophrenia-model AUC was 0.55 (accuracy = 48.97 %, sensitivity = 75.12 %, specificity = 49.80 %, PPV = 21.73 %, and NPV = 87.13 %). It is hard to draw direct comparisons with the original model due to the different sample sizes, but when the same predictors were selected by more than one model, the direction of effect was consistent (Table 4).

## 4 Discussion

The results of this study show that treatment resistance can be predicted with modest accuracy, using only clinical and demographic measures, when a patient with psychosis first presents to clinical services at the start of their illness. After correcting for optimism, the model was able to correctly identify 48 % of TR patients and 76 % of responders. 28 % of TR patients were correctly classified while 88 % of responders were correctly classified. This is the largest analysis of TR conducted to date; combining data from many research groups and driven by an international collaboration.

In our explanatory model, for each one-year decrease in age at onset there was an increase in the odds of developing TR. Previous research has found that younger age of onset is associated with both TR (Legge et al., 2020; Wimberley et al., 2016), a broad range of poor outcomes in schizophrenia (Immonen et al., 2017), and poor response to treatment in other disorders, including depression (Perlman et al., 2019) and bipolar (Hui et al., 2019). Immonen et al. (2017) suggest that younger age of onset results in poor outcomes because it disrupts social and cognitive development. There is evidence to support this claim: in a recent meta-analysis (Rajji et al., 2009), adult and adolescent-onset schizophrenia were both associated with cognitive deficits across multiple domains, but those with adolescent-onset schizophrenia had greater deficits on domains including IQ, executive functioning, and verbal memory. Adolescent-onset psychosis has also been associated with more negative symptoms at first-presentation (Downs et al., 2019) and worse premorbid functioning between the ages of 16 and 18 (Ballageer et al., 2005). Furthermore, cognitive deficits, particularly poor verbal memory (de Bartolomeis et al., 2013; Joober et al., 2002), an increased burden of negative symptoms (Iasevoli et al., 2018b), and worse premorbid functioning (Albert et al., 2011; Levine and Rabinowitz, 2010) have all been associated with non-response to antipsychotic treatment. In contrast, a small study of age of onset and TR found that TR explained most associations with symptom severity and functional impairment; only a few independent effects of age of onset were identified (one being poorer visuospatial memory in early onset TR participants) and the authors suggest that the associations observed between earlier age of onset and worse clinical outcomes could be an indirect effect of a TR subtype of schizophrenia (Iasevoli et al., 2022). Our finding that only younger age of onset and fewer years in education are associated with TR, after controlling for other factors, is in line with the hypothesis that TR has a neurodevelopmental aetiology. However, given that age of onset and years of education are independently associated with TR, there may be more than one path from disrupted cognitive and social development to TR.

Our prediction model automatically selected age of onset and years in education for inclusion in the model. Some of the other five predictors selected by the model (gender, BMI, relationship status, alcohol use, positive symptoms) have been previously associated with TR, but for the others their selection in the prediction model justifies further work in understanding their role in TR. Explanatory models of TR using longitudinal data have identified younger age of onset (Chan et al., 2021; Wimberley et al., 2016), being male (Szymanski et al., 1995), and never marrying (Emsley et al., 2006) as factors associated with TR. Previous studies comparing TR and NTR patients have failed to find a difference in the number of years in education (Frydecka et al., 2016; Iasevoli et al., 2018c), alcohol use (Chen et al., 2018), and positive symptoms (Freitas et al., 2019; Iasevoli et al., 2018a), although one study of treatment non-responders vs responders found a difference in BMI (Chiliza et al., 2015). It is important to note, however, that our model coefficients can be only interpreted in the context of their contribution to the prediction of TR. The coefficients are biased due to the penalization and are hence not an estimate of a population parameter.

The sensitivity and specificity of our prediction model suggests that, after external validation, it would not be suited for use in clinical settings due to the high misclassification rate, at least not as the sole decision aid for antipsychotic treatment which can have burdensome side effects. Nevertheless, the model as it stands would be more cost effective than current treatment practice in the UK. Jin et al. (2019) calculated the cost of implementing a prediction model for TR in clinical practice. They considered a test that could be used after patients had failed to respond to one antipsychotic. Those who were unlikely to respond to a second antipsychotic, according to the test, would be offered clozapine as a second-line treatment. Compared to treatment as usual (i.e. clozapine as a third-line treatment for all patients), and taking into consideration the side effects of first-line antipsychotics and clozapine, using a prediction model in this scenario would result in an improvement of 0.10 quality-adjusted life years (QALYs) and reduce healthcare costs by £7363 per person. Their analysis suggests that a predictive model would be more cost effective than treatment as usual even if it only accurately identified 6 % of TR patients and 50 % of NTR patients.

Although an AUC of 0.59 means the prediction model may not be of clinical use, it provides a base on which to improve stratified medicine for TR. The addition of other predictors may improve model performance and likely candidates include additional clinical data (Ajnakina et al., 2020; Legge et al., 2020), genetic risk for TR (Pardiñas et al., 2022), multiple polygenic risk scores for other traits (Krapohl et al., 2018), anterior cingulate cortex glutamate levels (Egerton et al., 2021), and lack of response in the first two weeks of treatment (Samara et al., 2015). It could also be useful in the context of randomized controlled trials; used to recruit samples enriched for TR (Vickers et al., 2006), added as a covariate (Hernández et al., 2004), or to stratify existing data for further analysis.

### 4.1 Strengths and limitations

Our study uses only data collected at first-episode, encompassing the full range of first episode presentations of psychosis. Many studies rely on cross-sectional or retrospective case-control designs which suffer from selection bias since they tend to include only patients who remain in mental health services and exclude those with good outcomes. Furthermore, cross-sectional or retrospective studies cannot exclude reverse causation. We cannot therefore assume that findings from these studies will generalise to first-episode patients.

One of the strengths of this analysis is that we have estimated, separately, the strength of associations, between characteristics at first-episode and TR, and the predictive accuracy of these same characteristics (Shmueli, 2010). A LASSO regression is an optimal method for prediction in this context. Variable selection is an automatic part of the model, which produces a parsimonious model (Kuhn and Johnson, 2013); although there is always a possibility that an important predictor is not selected, models with fewer predictors are easier to apply to new samples, since the amount of information required from participants is reduced, as well as to existing samples, as there is a higher chance that all the required data has been collected. LASSO regression combined with random forests imputation also prevents predictors selected by chance from having excess importance; this is important in our analysis since a limitation of the random forests imputation method is that it can give more weight to these false predictors when they have a large proportion of missingness and when the percentage of missingness varies across predictors (Lu and Petkova, 2014). LASSO handles multicollinearity well, a consistent occurrence in clinical samples, and we took the further step of removing highly correlated variables before any analyses. LASSO regressions have been shown to work well in clinical data sets (Kuhn and Johnson, 2013), and have good predictive accuracy when determining psychosis onset (Ciarleglio et al., 2018), schizophrenia diagnosis (Salvador et al., 2017), and response to clozapine (Fonseca de Freitas et al., 2022).

After acknowledging that our sample size may have limited the power of our analyses, the main limitations of our statistical methodology are that we have not been able to externally validate our prediction model (and split-sample validation is not recommended; Austin and Steyerberg, 2017), an important step in evaluating the clinical utility of a model (Kapur et al., 2012). An equally important limitation is the high proportion of missingness in our data; a consequence of combining multiple pre-existing cohorts. The percentage of missingness may have affected the imputations and introduced some bias to the model regression coefficients (Seijo-Pardo et al., 2019). In addition, variable selection for prediction models can be unreliable for predictors with a high proportion of missingness. For predictors with a high proportion of missingness, we cannot generalise that selected predictors are important or that non-selected predictors are not important; we can only conclude that they are important and not important for our specific prediction model after accounting for random forests imputation. A final limitation of our statistical methodology is that we did not allow for nonlinear predictors.

There are two key limitations of our participant sample: this is a sample of first-episode *psychosis* patients – not all of whom were later diagnosed with schizophrenia – and the rates of TR vary between the cohorts. We did not restrict the main analysis to those with a schizophrenia diagnosis to ensure we had the largest sample size possible for model development (not all cohorts recorded diagnosis at follow up). However, this is comparable to other work predicting TR (Legge et al., 2020) and may be more clinically useful since diagnosis is often unclear at first-episode; a recent meta-analysis found that patients eventually diagnosed with schizophrenia are often diagnosed with other psychotic disorders at first-episode (Fusar-Poli et al., 2016). Regardless, this may still have biased our sample and, as a consequence, we were not able to use the Treatment Response and Resistance in Psychosis (TRRIP) working group criteria for TR (Howes et al., 2017). As a result, different definitions of TR were used across different cohorts (see Supplementary Table 6), which may have led to the substantial variation in the rates of TR. According to a recent meta-analysis, the rate of treatment resistant schizophrenia – as defined by the TRRIP criteria – is estimated to be 22.8 % (95 % CI 19.1–27.0 %) among all first-episode cohorts and 24.4 % (95 % CI 19.5–30.0 %) among first-episode schizophrenia cohorts (Siskind et al., 2022). The Istanbul cohort was the only cohort to exclusively include participants with a diagnosis of schizophrenia, and its rate of TR (27.41 %) fell within the estimated 95 % CI for first-episode schizophrenia cohorts. Of the first-episode psychosis cohorts, only two cohorts were within the estimated 95 % CI (GAP London, 24.74 %, and AESOP London, 26.57 %). The Prague cohort had a substantially higher rate of TR (45.71 %), but notably this cohort was the only one where a measure of clinical response was not used. The West London cohort had the lowest rate of TR (3.70 %) and was the only cohort where TR was defined using clozapine only. The other five cohorts with lower rates of TR, than estimated in the meta-analysis, were the only cohorts to include a measure of functional response in the definition of TR (at least moderate functional impairment measured using the GAF or CGI scale), which may have proved too stringent a threshold when applied to secondary data analysis. Our prediction model may have had better accuracy if there was less measurement error in our outcome. The differing rates of TR reflect one of the statistical problems of international research consortia (Budin-Ljøsne et al., 2014): the violation of the assumption of homogeneity. Due to the proportion of missing data in our sample, we were not able to use methods such as leave-one-cohort-out cross validation or meta-analyses to estimate the heterogeneity. However, Bühlmann and van de Geer (2018) argue that sample heterogeneity can produce more robust estimates in causal modelling and while heterogeneity is a problem for robust prediction, our model still discriminated between TR and NTR cases better than chance given all the described problems.

### 4.2 Conclusion

Our results show that younger age of onset and fewer years in education at first-episode of psychosis are independently associated with TR. We have developed and internally validated a parsimonious prediction model for TR. This model can quantify future individuals' probability of developing TR with modest predictive accuracy and using only clinical and demographic information recorded at first-episode. We have identified novel predictors of TR (BMI, alcohol use, years in education, positive symptoms) and confirmed existing predictors (age of onset, gender, relationship status). At present, this is the largest sample of prospective psychosis patients where TR status is known. Further work is required to build a clinically useful model for predicting TR; but there is scope to develop a new model using our identified predictors and other known predictors from the wider literature.

## Data availability

Data can be requested by contacting james.maccabe@kcl.ac.uk and completing a STRATA secondary analysis proposal form which will then be circulated to all consortium members for review.

## Twitter

NEW prediction model for treatment resistance in schizophrenia, secondary data-analysis of prospective first-episode cohorts, good with lasso, seeking like-minded researchers to replicate and update. GSOH needed to read this tweet.

## Funding

This work was supported by a Stratified Medicine Programme grant to JHM from the 10.13039/501100000265Medical Research Council (grant number MR/L011794/1 which funded the research and supported S.E.S., D.A., A.F.P, L.K., R.M.M., D.S., J.T.R.W, & J.H.M.); funding from the National Institute for Health Research Biomedical Research Centre at South London and Maudsley National Health Service Foundation Trust and King's College London to D.A. and D.S; and funding from the Collaboration for Leadership in Applied Health Research and Care (CLAHRC) South London at King's College Hospital National Health Service Foundation Trust to S.E.S.

The views expressed are those of the author(s) and not necessarily those of the Medical Research Council, National Health Service, the National Institute for Health Research, or the Department of Health.

The AESOP (London, UK) cohort was funded by the UK Medical Research Council (Ref: G0500817). The Belfast (UK) cohort was funded by the Research and Development Office of Northern Ireland. The Bologna (Italy) cohort was funded by the European Community's Seventh Framework Program under grant agreement (agreement No. HEALTH-F2-2010–241909, Project EU-GEI). The GAP (London, UK) cohort was funded by the UK National Institute of Health Research (NIHR) Specialist Biomedical Research Centre for Mental Health, South London and Maudsley NHS Mental Health Foundation Trust (SLaM) and the Institute of Psychiatry, Psychology, and Neuroscience at King's College London; Psychiatry Research Trust; Maudsley Charity Research Fund; and the European Community's Seventh Framework Program grant (agreement No. HEALTH-F2-2009-241909, Project EU-GEI). The Lausanne (Switzerland) cohort was funded by the Swiss National Science Foundation (no. 320030_135736/1 to P.C. and K.Q.D., no 320030-120686, 324730-144064 and 320030-173211 to C.B.E and P.C., and no 171804 to LA); National Center of Competence in Research (NCCR) “SYNAPSY - The Synaptic Bases of Mental Diseases” from the Swiss National Science Foundation (no 51AU40_125759 to PC and KQD); and Fondation Alamaya (to KQD). The Oslo (Norway) cohort was funded by the Research Council of Norway (#223273/F50, under the Centers of Excellence funding scheme, #300309, #283798) and the South-Eastern Norway Regional Health Authority (#2006233, #2006258, #2011085, #2014102, #2015088 to IM, #2017-112). The Paris (France) cohort was funded by European Community's Seventh Framework Program grant (agreement No. HEALTH-F2-2010–241909, Project EU-GEI). The Prague (Czech Republic) cohort was funded by the Ministry of Health of the Czech Republic (Grant Number: NU20-04-00393). The Santander (Spain) cohort was funded by the following grants (to B.C.F): Instituto de Salud Carlos III, FIS 00/3095, PI020499, PI050427, PI060507, Plan Nacional de Drogas Research Grant 2005-Orden sco/3246/2004, and SENY Fundatio Research Grant CI 2005-0308007, Fundacion Marques de Valdecilla A/02/07 and API07/011. SAF2016-76046-R and SAF2013-46292-R (MINECO and FEDER). The West London (UK) cohort was funded The Wellcome Trust (Grant Number: 042025; 052247; 064607).

## CRediT authorship contribution statement

S.E.S., D.A., A.F·P., R.M.M., J.T.R.W., D·S., and J.M. designed the study, interpreted the data, and revised the manuscript. S.E.S. acquired the data for secondary data analysis. S.E.S. and D.A. conducted the analysis. S.E.S. drafted the manuscript. All other authors contributed to the interpretation of the data and revised the manuscript.

## Declaration of competing interest

The Authors declare no Competing Non-Financial Interests but the following Competing Financial Interests: J.T.R.W. is an investigator on a grant from Takeda Pharmaceuticals Ltd. to Cardiff University, for a project unrelated to the work presented here. S.E.S. is employed on this grant. M.D.F. has received a fee for educational seminars from Lundbeck and Janssen. O.A.A. is a consultant to HealthLytix and has received speakers honorarium from Lundbeck and Sunovion. T.R.E.B. has been a member of an advisory board for Gedeon Richter. C.B.E. received honoraria for conferences from Forum pour la formation médicale, Janssen-Cilag, Lundbeck, Otsuka, Sandoz, Servier, Sunovion, Sysmex Suisse AG, Takeda, Vifor-Pharma, and Zeller in the past 3 years. B.C.F. has received honoraria for participation as a consultant and/or as a speaker at educational events from ADAMED, Mylan, Angelini, Janssen Johnson & Johnson, Lundbeck, and Otsuka Pharmaceuticals. R.M.M. has received payments for non-promotional lectures from Janssen, Otsuka, Sunovian, and Lundbeck. J.H.M. has received research funding from H Lundbeck.

## Appendix A Supplementary data

Supplementary material Image 1
