# Supplementary material for: Clinical predictors of antipsychotic treatment resistance: Development and internal validation of a prognostic prediction model by the STRATA-G consortium
Source: Schizophr Res. 2022 Dec;250:1–9. doi: 10.1016/j.schres.2022.09.009 (PMC9834064; doi:10.1016/j.schres.2022.09.009)
Supplement: Supplementary file 1 — Supplementary material [file mmc1.docx]

# Clinical Predictors of Antipsychotic Treatment Resistance: Development and internal validation of a prognostic prediction model by the STRATA-G Consortium

**Supplementary Material**

Table of Contents

1. TRIPOD Checklist: Prediction Model Development and Validation 3

2. A Description of the STRATA-G Dataset 5

3. Cohorts 5

4. King’s College London (London, UK) 5

7. Queen’s University Belfast (Belfast, UK) 5

10. University of Bologna (Bologna, Italy) 5

13. Istanbul University (Istanbul, Turkey) 6

14. Lausanne University Hospital (Lausanne, Switzerland) 6

15. University of Oslo (Oslo, Norway) 6

16. French Institute of Health and Medical Research (Paris, France) 6

17. National Institute of Mental Health (Prague, Czech Republic) 6

18. Marqués de Valdecilla University Hospital (Santander, Spain) 6

19. University of São Paulo (São Paulo, Brazil) 7

20. University College London and Imperial College London (London, UK) 7

21. Defining Treatment Resistance 7

22. Predictors 7

23. Accommodation 8

24. Age at baseline 8

25. Age at onset 8

26. Alcohol 8

27. Body Mass Index (BMI) 8

28. Brief Psychiatric Rating Scale (BPRS) 9

29. Cannabis 9

30. Duration of untreated psychosis (DUP) 9

31. Education qualifications 9

32. Employment 9

33. Ethnicity 9

34. Family history of mental health disorders 9

35. Family history of psychosis 9

36. Gender 10

37. Global Assessment of Functioning (GAF) 10

38. Living situation 10

39. Positive and Negative Syndrome Scale (PANSS) 10

40. Relationship status 10

41. Scale for the Assessment of Negative Symptoms (SANS) 10

42. Scale for the Assessment of Positive Symptoms (SAPS) 11

43. Tobacco 11

44. Years in education 11

45. Supplementary Methods 12

46. Data Preparation 12

47. Explanatory Model 12

48. Prediction Model 12

49. Supplementary Results 13

50. Explanatory Model 13

51. Missing data imputation 13

52. Prediction Model 13

53. Missing data imputation 13

54. Lambda and threshold 13

55. Performance measures 13

56. Equation 14

57. References 15

58. Supplementary Tables & Figures 20

# TRIPOD Checklist: Prediction Model Development and Validation

| **Section/Topic** | **Item** |  | **Checklist Item** | **Page** |
| --- | --- | --- | --- | --- |
| **Title and abstract** | | | | |
| Title | 1 | D;V | Identify the study as developing and/or validating a multivariable prediction model, the target population, and the outcome to be predicted. | Main Title |
| Abstract | 2 | D;V | Provide a summary of objectives, study design, setting, participants, sample size, predictors, outcome, statistical analysis, results, and conclusions. | Main Abstract |
| **Introduction** | | | | |
| Background and objectives | 3a | D;V | Explain the medical context (including whether diagnostic or prognostic) and rationale for developing or validating the multivariable prediction model, including references to existing models. | Main Intro |
|  | 3b | D;V | Specify the objectives, including whether the study describes the development or validation of the model or both. | Main Intro |
| **Methods** | | | | |
| Source of data | 4a | D;V | Describe the study design or source of data (e.g., randomized trial, cohort, or registry data), separately for the development and validation data sets, if applicable. | Main Methods |
|  | 4b | D;V | Specify the key study dates, including start of accrual; end of accrual; and, if applicable, end of follow-up. | Supp 5-7 |
| Participants | 5a | D;V | Specify key elements of the study setting (e.g., primary care, secondary care, general population) including number and location of centres. | Supp 5-7 |
|  | 5b | D;V | Describe eligibility criteria for participants. | Main Methods |
|  | 5c | D;V | Give details of treatments received, if relevant. | Main Methods  Supp 7-11 |
| Outcome | 6a | D;V | Clearly define the outcome that is predicted by the prediction model, including how and when assessed. | Main Methods  Supp 7-11 |
|  | 6b | D;V | Report any actions to blind assessment of the outcome to be predicted. | None |
| Predictors | 7a | D;V | Clearly define all predictors used in developing or validating the multivariable prediction model, including how and when they were measured. | Main Methods  Supp 11-15 |
|  | 7b | D;V | Report any actions to blind assessment of predictors for the outcome and other predictors. | None |
| Sample size | 8 | D;V | Explain how the study size was arrived at. | Main Methods  Supp 15  Supp Table 1, 2 |
| Missing data | 9 | D;V | Describe how missing data were handled (e.g., complete-case analysis, single imputation, multiple imputation) with details of any imputation method. | Main Methods  Supp 15-18 |
| Statistical analysis methods | 10a | D | Describe how predictors were handled in the analyses. | Main Methods  Supp 15-18 |
|  | 10b | D | Specify type of model, all model-building procedures (including any predictor selection), and method for internal validation. | Main Methods  Supp 15, 18-21 |
|  | 10c | V | For validation, describe how the predictions were calculated. | Supp 21 |
|  | 10d | D;V | Specify all measures used to assess model performance and, if relevant, to compare multiple models. | Main Methods |
|  | 10e | V | Describe any model updating (e.g., recalibration) arising from the validation, if done. | Main Methods |
| Risk groups | 11 | D;V | Provide details on how risk groups were created, if done. | NA |
| Development vs. validation | 12 | V | For validation, identify any differences from the development data in setting, eligibility criteria, outcome, and predictors. | NA |
| **Results** | | | | |
| Participants | 13a | D;V | Describe the flow of participants through the study, including the number of participants with and without the outcome and, if applicable, a summary of the follow-up time. A diagram may be helpful. | Main Methods  Supp 15 |
|  | 13b | D;V | Describe the characteristics of the participants (basic demographics, clinical features, available predictors), including the number of participants with missing data for predictors and outcome. | Table 2 |
|  | 13c | V | For validation, show a comparison with the development data of the distribution of important variables (demographics, predictors and outcome). | NA |
| Model development | 14a | D | Specify the number of participants and outcome events in each analysis. | Main Results Table 1 |
|  | 14b | D | If done, report the unadjusted association between each candidate predictor and outcome. | Supp Table 5 |
| Model specification | 15a | D | Present the full prediction model to allow predictions for individuals (i.e., all regression coefficients, and model intercept or baseline survival at a given time point). | Supp 21  Supp Table 5 |
|  | 15b | D | Explain how to the use the prediction model. | Supp 21 |
| Model performance | 16 | D;V | Report performance measures (with CIs) for the prediction model. | Supp 19-21 |
| Model-updating | 17 | V | If done, report the results from any model updating (i.e., model specification, model performance). | NA |
| **Discussion** | | | | |
| Limitations | 18 | D;V | Discuss any limitations of the study (such as nonrepresentative sample, few events per predictor, missing data). | Main Discussion |
| Interpretation | 19a | V | For validation, discuss the results with reference to performance in the development data, and any other validation data. | NA |
|  | 19b | D;V | Give an overall interpretation of the results, considering objectives, limitations, results from similar studies, and other relevant evidence. | Main Discussion |
| Implications | 20 | D;V | Discuss the potential clinical use of the model and implications for future research. | Main Discussion |
| **Other information** | | | | |
| Supplementary information | 21 | D;V | Provide information about the availability of supplementary resources, such as study protocol, Web calculator, and data sets. | Main Methods, Data Availability Statement |
| Funding | 22 | D;V | Give the source of funding and the role of the funders for the present study. | Main Funding |

# A Description of the STRATA-G Dataset

## Cohorts

### King’s College London (London, UK)

#### AESOP

The AESOP study (Aetiology and Ethnicity in Schizophrenia and Other Psychoses) is a multi-centre, naturalistic, prospective incidence and case-control study of first episode psychosis, conducted initially over a three-year period from September 1997 to August 2000. The study sample comprises: patients with an International Statistical Classification of Diseases and Related Health Problems, Tenth Revision ICD-10; ^1^ diagnosis of F10-F29 or F30-F33 (psychosis codes), aged 16-65 years, who presented to secondary and tertiary services within tightly defined catchment areas in south-east London, Nottingham, and Bristol ^2-7^. All participants, in centres in southeast London and Nottingham, UK, were invited to take part in a follow-up study, at approximately 10 years after baseline ^8-10^ and those without bloods samples were invited to take part in a follow-up approximately 15 years after baseline for STRATA workstream three (WS3). Treatment resistance/non-resistance was determined by Dr Arsime Demjaha^8^ and Dr Sophie Smart.

#### GAP

The GAP study (Genetics and Psychosis) is a population-based incidence and case-control study of first episode psychosis, conducted initially over a five-year period from December 2005 to December 2010. The study sample comprises: patients with a ICD-10 diagnosis of F20.0, F25.0, F28.0, F29.0 (verified by the SCAN), aged 18-65 years, who had been admitted to psychiatric inpatient units or seen by community-based mental health teams within the South London and Maudsley (SLaM) NHS Foundation Trust^11,12^. The study exclusion criteria were evidence of 1) psychotic symptoms precipitated by an organic cause; 2) evidence of transient psychotic symptoms resulting from acute intoxication as defined by ICD-10; 3) moderate or severe learning disabilities as defined by ICD-10; or 4) head injury causing clinically significant loss of consciousness. Approximately 5 years after first contact for psychosis^13,14^, information at follow-up was collated from the electronic psychiatric record-keeping system within the SLaM Trust^15^ using the WHO Life Chart Schedule extended version^16^. Treatment resistance/non-resistance was determined by Dr Olesya Ajnakina^13^.

### Queen’s University Belfast (Belfast, UK)

#### NIFEPS

The Belfast data is from two studies. The NIFEPS study (Northern Ireland First Episode Psychosis) is a naturalistic, prospective, incidence study, conducted initially over a two-year period from January 2003 and December 2004. The study sample comprises: patients with an Operational Criteria checklist for Psychotic Illness OPCRIT; ^17^ diagnosis of first episode psychosis, aged 18–64 years, and living in Northern Ireland. All participants were invited to take part in follow-up studies, 1 year after baseline ^18^ and approximately 13 years after baseline as part of STRATA WS3.

#### RGPI

The RGPI study (Resources for Genomics, Ireland) is a multi-centre, population-based, incidence study of first episode psychosis conducted from 2007. The study sample comprises: patients with a Diagnostic Statistical Manual version four DSM-IV; ^19^ diagnosis of schizophrenia, schizophreniform disorder, schizoaffective disorder or bipolar affective disorder with psychosis, aged 16+ years, who had Irish born grandparents, and who presented to psychiatric services in the region of the research centres ^20^. All participants, recruited through psychiatric services in the region of Queen’s University, Belfast, were invited to take part in a follow-up study, at approximately 9 years after baseline as part of STRATA WS3. Treatment resistance/non-resistance, in both samples, was determined by Dr Lina Homman.

### University of Bologna (Bologna, Italy)

#### EUGEI

The Bologna data is from two studies. EUGEI (European Network of National Schizophrenia Networks Studying Gene-Environment Interactions) is a multi-centre, population-based incidence and case-sibling-control study of first episode psychosis conducted initially, in Bologna, over a four-year period from January 2011 to December 2014. The study sample comprises: patients with a ICD-10 diagnosis of F20-F33, aged 18-64 years, who presented to services within the catchment area ^21^. All participants, in Bologna, were invited to take part in a follow-up study, in 2016.

#### BoFEP

The BoFEP study (Bologna FEP) is an ongoing, naturalistic, prospective incidence study of first episode psychosis, conducted initially over an eight-year period from January 2002 and December 2009. The study sample comprises: patients with an ICD-10 diagnosis of F10–F29 or F30–F33, aged 18-64 years, who presented to services within the defined catchment area in West Bologna. All participants were invited to take part in a follow-up study, 1 year after baseline ^22^. Treatment resistance/non-resistance, in both samples, was determined by Dr Lina Homman.

### Istanbul University (Istanbul, Turkey)

The Istanbul data is from an ongoing, hospital-based incidence study of first episode schizophrenia, conducted from 1996. This study is sometimes known as the First-Episode Schizophrenia Follow-Up Project and a proportion of this sample was included in EUGEI. The study sample comprises: patients with a DSM-IV diagnosis of schizophrenia, aged 15-45 years, who were experiencing an acute phase of their first psychotic episode and being treated as an inpatient ^23-26^. All participants were invited to take part in a follow-up study, 2+ year after their baseline ^23^. Treatment resistance/non-resistance was determined by Dr Alp Üçok.

### Lausanne University Hospital (Lausanne, Switzerland)

The TIPP study (Treatment and Early Intervention in Psychosis Program) is an ongoing, naturalistic prospective study of early onset psychosis, initiated and conducted by Dr Philippe Conus from 2004. The study sample comprises: patients who meet threshold criteria for psychosis (defined by the ‘Psychosis threshold’ subscale of the Comprehensive Assessment of At Risk Mental States scale CAARMS; ^27^), aged 18-35 years, who reside in the Lausanne catchment area ^28-30^. All participants enrolled in TIPP are invited to take part in follow-up studies, lasting 3 years after baseline. A subsample of TIPP patients were included in STRATA-G: those that participated either in a neurobiological research study developed by Prof Kim Do ^30^, and/or were part of PsyMetab or Psyclin studies led by Dr Chin Eap ^31-35^. Treatment resistance/non-resistance was determined by Dr Romeo Restellini, Dr Luis Alameda, and Dr Sara Camporesi.

### University of Oslo (Oslo, Norway)

The TOP study (Thematic Organized Psychosis Research) is a naturalistic, prospective incidence and case-control study of first episode psychosis, conducted initially over a twelve-year period from May 2003 to 2015. The study sample comprises: patients with a DSM-IV diagnosis of schizophrenia, schizophreniform disorder, schizoaffective disorder, psychosis not otherwise specified (NOS), delusional disorder, brief psychosis or major affective disorder with mood incongruent psychotic symptoms, aged 18-65 years, within 1 year of the start of their first adequate treatment with antipsychotic medication, who presented to outpatient and inpatient services within four University Hospitals in Oslo ^36,37^. All participants were invited to take part in a follow-up study, approximately 1 year after baseline ^38-40^. Treatment resistance/non-resistance was determined by Dr Carmen Simonsen and Professor Ingrid Melle.

### French Institute of Health and Medical Research (Paris, France)

As part of the EUGEI, a multi-centre, population-based incidence and case-sibling-control study of first episode psychosis, subjects were assessed initially, in Créteil and Paris, over a two-year period from December 2010 to June 2014. The study sample comprises: patients with a ICD-10 diagnosis of F20-F33, aged 18-64 years, who presented to services within the catchment area ^21^. All participants, in Créteil and Paris, were invited to take part in a follow-up study, in 2017. Treatment resistance/non-resistance was determined by Dr Andrei Szöke, Aziz Ferchiou, Baptiste Pignon, Andrea Tortelli, and Jean-Romain Richard.

### National Institute of Mental Health (Prague, Czech Republic)

The Early Stages of Schizophrenia study is a hospital-based incidence study of first episode schizophrenia, conducted initially over an unreported period of time. The study sample comprises: patients with a ICD-10 diagnosis of F20 or F23, aged 18-35 years, who had less than 2 years of untreated psychosis, and who were hospitalised in a large general psychiatry hospital that serves Prague and part of Central Bohemia regions ^41-44^. All participants were invited to take part in a follow-up study, 1 year after baseline. Treatment resistance/non-resistance was determined by Dr Lina Homman.

### Marqués de Valdecilla University Hospital (Santander, Spain)

The PAFIP study (First Episode Psychosis Clinical Program) is an ongoing, naturalistic, prospective incidence study of first episode psychosis, conducted from February 2001. The study sample comprises: patients with an DSM-IV diagnosis of non-affective psychosis, aged 15+ years, who were referred from mental health services in the region of Cantabria. All participants were invited to take part in a follow-up studies, 3+ years after baseline ^45-48^. Treatment resistance/non-resistance was determined by Dr. Benedicto Crespo-Facorro and Dr Javier Vázquez-Bourgon.

### University of São Paulo (São Paulo, Brazil)

The Brazilian Wellcome Trust sample is a naturalistic, prospective incidence study of first episode psychosis, conducted between July 2002 and December 2004. The study sample comprises: patients with an DSM-IV diagnosis for psychotic disorder, aged 18-64 years, who had a first contact with mental health services due to a psychotic episode and who had been living in the defined geographical region of Sao Paulo for at least six months ^49-53^. Participants who took part in the MRI part of the baseline study were invited to take part in a follow-up study, 2 years after baseline ^52^. Treatment resistance/non-resistance was determined by Dr Sophie Smart.

### University College London and Imperial College London (London, UK)

The West London Longitudinal First-Episode Psychosis Study a naturalistic, prospective incidence study of first episode psychosis, conducted from 1998 to 2008. The study sample comprises: patients with an DSM-IV diagnosis of psychosis, aged 16-50 years, who were presenting with a psychotic illness for the first time and who had been receiving antipsychotic medication for less than 12 weeks. All participants were invited to take part in follow-up studies, 1, 3 and 5 years after baseline^54-62^. Treatment resistance/non-resistance was determined by Dr Sophie Smart.

## Defining Treatment Resistance

Treatment resistance (TR) was defined in two ways; participants could meet either of these two criteria to be classified as TR.

1. Lifetime clozapine treatment was used as a simple marker of treatment resistance. This included patients who reported being prescribed or taking clozapine at a study visit or patients whose clinical notes stated that they were prescribed clozapine.
2. The consensus definition developed by the *Treatment Response and Resistance in Psychosis (TRRIP) working group* ^63^.

The number of TR and non-treatment resistance (NTR) cases within each cohort are reported in Supplementary Table 6. In two cohorts only lifetime clozapine treatment was available (Sao Paulo and West London). As we are using legacy data there are instances when the criteria could not be applied as intended (e.g. in one cohort, their battery of tests did not include a measure of functioning). The grid below shows how the TRRIP criteria were applied to each cohort. TR criteria had already been applied to AESOP and GAP so these definitions were retained as they were comparable to the TRRIP criteria ^8,13^. For two cohorts clozapine use at baseline was reported (Belfast N=5 and West London N=3) and we removed these individuals from the database.

Our criteria deviated from the TRRIP criteria in three main ways; we did not apply their criteria concerning diagnosis, adherence, and symptom domain. Firstly, a confirmed diagnosis of schizophrenia was not included in the criteria for this study. Diagnosis at follow-up is not routinely collected in longitudinal studies and in our data 51% of follow-up diagnoses were missing. Only 25% of diagnoses at baseline were missing but in cohort studies, with only one study visit at baseline, diagnoses may not be valid. For example, DSM 5 criteria for schizophrenia require a disturbance of six months and active symptoms for one month, so this diagnosis cannot be applied to participants who have been ill for less than six months at the time of the baseline assessment. Secondly, to be consistent across cohorts, patients did not need to be adherent to antipsychotics to meet the TRRIP definition of TR, as only two cohorts recorded adherence. In the Santander cohort, a subjective measure of adherence at one and three-year follow-ups was recorded (good vs. poor adherence). In the Lausanne cohort, antipsychotic blood levels were recorded, however this data was not used to determine TR status. Thirdly, we did not specify which subclinical domain persistence symptoms must fall into. The TRRIP working group stipulate the need for subclinical specifiers (e.g. “positive,” “negative,” or “cognitive”). Not using subclinical specifiers means our findings are comparable to research published before the TRRIP guidelines.

## Predictors

Any variables recorded in more than one cohort, at baseline, were considered as potential predictors. Details of how these variables were defined are below and descriptive statistics, along with the proportion of missing data, is presented in Table 2 and Supplementary Table 1.

In addition, we calculated length of follow-up. Length of follow-up was recorded in years for GAP and Istanbul. However, for all other cohorts, length of follow was not recorded and, therefore, the difference between the baseline date and the furthest known follow-up date was calculated. This resulted in a length of follow-up measured in days, which was converted to years by dividing values by 365.25. Missing data was replaced with the average follow up for each cohort. For Bologna, follow-up dates were not recorded, but the original researchers stated that all follow-ups occurred over a relatively short time period and that the 15 Nov 2016 could be used as a proxy follow-up date for all participants.

We also noted whether participants had a diagnosis of schizophrenia at their last known follow up visit. When multiple diagnoses were recorded for follow up visit, ‘International Statistical Classification of Diseases and Related Health Problems’ 10th Edition (ICD-10) diagnoses were given priority over ‘Diagnostic and Statistical Manual of Mental Disorders’ 4th Edition (DSM-IV) diagnoses, which were in turn given priority over diagnoses using unspecified criteria.

All the below variables were collected at baseline unless explicitly stated otherwise.

### Accommodation

Accommodation status at baseline was recorded in Bologna, Lausanne, Oslo, and Paris, however, each used a different ordinal scale to capture this data. From this information, we created a binary variable: in supported accommodation vs. independent living. We considered supported accommodation to include both practical support (e.g. assisted living, supervised living, institutions, care home) and financial support (e.g. council housing). We classified living with family as independent living; we did not have enough data to distinguish between participants living with family due to their illness or for other reasons (e.g. student or carer).

### Age at baseline

Participant’s age at the time of the baseline assessment was available for all samples.

### Age at onset

Age of onset was considered to be the participant’s age when psychotic symptoms first occurred. Age of first psychotic symptoms was recorded at baseline for AESOP, Istanbul, Lausanne, Oslo, and Santander. If this variable was not available, the participant’s age when they first presented to clinical services for psychosis was used. Age of first presentation to clinical services for psychosis was recorded at baseline for Belfast, GAP, and Paris. Age of first presentation to clinical services for psychosis was recorded at the five year follow-up for Bologna and since this is considered a static trait we included this data. As date of first presentation to clinical services is likely to be systematically later than date of first psychotic symptoms, we applied a correction to age at first presentation to clinical services when it was used instead of true age of onset. One cohort included in STRATA-G, AESOP, collected data on both age at first presentation and age of first symptoms. The mean difference between these ages was 0.547 years. Therefore, the estimated age of onset, in the absence of the variable, equalled age at first presentation to clinical services minus 0.547 years.

### Alcohol

Alcohol use at baseline was recorded in Belfast, Lausanne, Santander, and West London, however each used a different scale. From this information, we created a binary variable: non-drinker vs. drinker. Alcohol use (yes vs. no) was recorded at baseline in Santander. DSM-IV criteria for alcohol use was recorded in Belfast (five categories: never used, abstention, use, abuse, and dependence). Never used and abstention were classified as ‘non-drinker’, while use, abuse, and dependence were classified as ‘drinker’. The Case Manager Rating Scale CMRS; ^64^ for alcohol use was recorded in Lausanne (four categories: absent, light, moderate, and severe). Absent was classified as ‘non-drinker’, while light, moderate and severe were classified as ‘drinker’. Alcohol use in the last six months, measured in number of units, was recorded in Oslo. Zero units in the last six months was classified as ‘non-drinker’, while more than zero units in the last six months was classified as ‘drinker’. Alcohol use was also recorded in West London (three categories: no, yes, and dependent). No was classified as ‘non-drinker’, while yes and dependent were classified as ‘drinker’.

### Body Mass Index (BMI)

BMI at baseline was recorded in Oslo and Santander. Weight in kilograms (kg) and height in centimetres (cm) at baseline was recorded in Belfast and GAP. The following formula was used to calculate BMI in Belfast and GAP: (weight/(height/100))/(weigh/100).

### Brief Psychiatric Rating Scale (BPRS)

BPRS scores were recorded in Istanbul and Santander. The BPRS is designed to measure psychiatric symptoms, including hallucinations, depression, anxiety, and usual behaviour, across 24 items using a 1-7 Likert scale (1 equates ‘not present’ and 7 equates to ‘extremely severe’) ^65^. The minimum score on the BPRS is 24 while the maximum is 168.

### Cannabis

Cannabis use at baseline was recorded in Belfast, Bologna, GAP, Lausanne, Oslo, Santander, and West London, however each used a different scale. From this information, we created a binary variable: cannabis vs. no cannabis. Cannabis use (yes vs. no) was recorded at baseline in Bologna, GAP, Santander, and West London. DSM-IV criteria for cannabis use was recorded in Belfast (five categories: never used, abstention, use, abuse, and dependence). Never used and abstention were classified as ‘no cannabis’, while use, abuse, and dependence were classified as ‘cannabis’. The CMRS for cannabis use was recorded in Lausanne (four categories: absent, light, moderate, and severe). Absent was classified as ‘no cannabis’, while light, moderate and severe were classified as ‘cannabis’. Cannabis use in the last six months (yes vs. no) was recorded in Oslo. No use in the last six months was classified as ‘no cannabis’, while use in the last six months was classified as ‘cannabis’.

### Duration of untreated psychosis (DUP)

DUP is the time in days between the first occurrence of psychotic symptoms and the start of antipsychotic treatment for psychosis. DUP was recorded in AESOP, Lausanne, Oslo, Paris, Santander, and West London. DUP measured in weeks (Oslo, Paris, and West London) was converted to days by multiplying by seven. DUP measured in months (West London) was converted to days by multiplying by 30.417.

### Education qualifications

Highest educational qualification was recorded at baseline for AESOP, Bologna, GAP, Istanbul, Paris, and Prague. For this analysis, we used four categories to define highest educational qualification: None (e.g. no qualifications, primary school, school without qualification), Basic (e.g. GCSE, school with qualifications, O levels, junior/high school, secondary school), Further (e.g. first level on non-compulsory education, A levels, high school, Baccalaureate, vocation or college BTEC, NVQ, technical college), and Higher (university, undergraduate degree, postgraduate degree, professional).

### Employment

Employment circumstances at baseline were recorded in Belfast, Bologna, GAP, Istanbul, Oslo, and Paris, however, each used a different ordinal scale. From this information, we created a binary variable: employed vs. unemployed. We considered ‘unemployed’ to include retired, economically inactive, rehabilitation welfare, disability benefit, and sick leave. Students were considered as ‘employed’ because in Istanbul and Oslo studying and working were grouped together.

### Ethnicity

Ethnicity was recorded at baseline for all cohorts, apart from Belfast. Ethnicity in Belfast was recorded at the 10-year follow-up assessment. As this is a static trait, we used follow-up ethnicity in lieu of baseline ethnicity. For this analysis, we used three categories to define ethnicity: European, (e.g. White British, White other, White UK, White Irish, White Italian, White East European, Caucasian, European, American, and Gipsy), Black (e.g. Black Caribbean, Black African, Maghreb, African, Black), and Asian/Mixed/Other (e.g. Asian, Mixed Black, Mixed Other, Other, White and Black African, Arab, Filipino, Chinese, Bangladeshi, Indian, Middle East, Hispanic, and Latin American).

### Family history of mental health disorders

Family history of mental health disorders (yes vs. no) was recorded in AESOP and Belfast. Parental and family history of any mental health disorder was recorded in AESOP, using the Family Interview for Genetic Studies (FIGS)^66^ and. Only family history of any mental health disorder, using the FIGS, was recorded in Belfast. Within this variable we also included family history of psychosis, as described under ‘Family history of psychosis’. Again, this included all known family members, and not just first-degree relatives.

### Family history of psychosis

Family history of psychosis (yes vs. no) was recorded in AESOP and West London. Parental and family history of psychosis was recorded in AESOP, using the FIGS and family history of schizophrenia was recorded in West London, using the Diagnostic Interview for Psychosis (DIP)^67^. This included all known family members, and not just first-degree relatives.

### Gender

For all cohorts, gender was a binary variable and participants were either categorised as male or female.

### Global Assessment of Functioning (GAF)

GAF scores were recorded in Belfast, GAP, Istanbul, Lausanne, Oslo, Paris, and Prague. The GAF is a scale, included in the DSM-IV, which is used to assess social, occupational, and psychological functioning^19^. Individuals are given a score from 100 (extremely high functioning) to 1 (severely impaired). The GAF is often rated by focusing on symptoms (GAF-S) only or on functioning/disability (GAF-F) only; the GAF, as a single score, is the most severe of the GAF-S and GAF-F. GAF, as a single score, was recorded in Belfast, Istanbul, Lausanne, and Prague. In GAP, Oslo, and Paris, the GAF was recorded as two scores, and for each individual the most severe score was used. In Lausanne, the GAF was recorded two months after the baseline assessment, but we chose to treat it as the baseline score.

### Living situation

Living circumstances at baseline were recorded in AESOP, Belfast, Bologna, GAP, Lausanne, Oslo, and Santander, however, each used a different ordinal scale to capture this data. From this information, we created a three-level variable: living alone, living with family (e.g. partner, children, parents, other family), and living with others (e.g. shared housing, friends). A variable from Santander which grouped living alone without children and living alone with children together was categorised as living alone.

### Positive and Negative Syndrome Scale (PANSS)

PANSS scores were recorded in Belfast, GAP, Lausanne, Oslo, and Prague. The PANSS is designed to measure symptoms of schizophrenia across 30 items using a 1-7 Likert scale for each domain (1 equates to ‘absent’ and 7 to ‘extreme’)^68^. The minimum score on the PANSS is 30 while the maximum is 210. There are three subscales within the PANSS. The PANSS positive symptom subscale is used to rate positive symptoms of schizophrenia across seven domains e.g. delusions, hallucinations, etc. The scores for each item are summed so that the minimum score is 7 (all symptoms are absent) and the maximum score is 49 (all symptoms are present and extreme). The PANSS negative symptom subscale is used to rate negative symptoms of schizophrenia across seven domains e.g. blunted affect, stereotyped thinking, etc. The scores for each item are summed so that the minimum score is 7 and the maximum score is 49. The PANSS general psychopathy symptoms subscale is used to rate symptoms that are not covered by the positive or negative subscales. This subscale covers 16 domains e.g. somatic concerns, anxiety, depression, lack of judgement and insight, etc. The scores for each item are summed so that the minimum score is 16 and the maximum score is 112.

### Relationship status

Relationship status at baseline were recorded in AESOP, Belfast, Bologna, GAP, Istanbul, Lausanne, Oslo, Paris, Prague, and Santander, however, each used a different ordinal scale to capture this data. From this information, we created two binary variables: current relationship and lifetime relationship; in a relationship vs not in a relationship (e.g. married, steady relationship, cohabiting, civil partnership vs. single, separated, divorced, widowed, never married and not cohabiting) and ever been in a relationship vs. never been in a relationship (e.g. married, steady relationship, cohabiting, civil partnership, separated, divorced, widowed vs. single, never married and not cohabiting)

### Scale for the Assessment of Negative Symptoms (SANS)

SANS scores were recorded in Belfast, Istanbul, Santander, and West London. The SANS is used to rate negative symptoms in the following five domains: flat affect, alogia, apathy, anhedonia, and attention^69,70^. Within each domain there are a number of individual items and one global item. Each item is rated on a 0-5 Likert scale (0 equates ‘absent’ and 5 equates to ‘severe’). The SANS composite total is a sum of all SANS items apart from the global items (items: 1-7, 9-12, 14-16, 18-21, and 23-24) the minimum score is 0 and the maximum score is 100. The SANS global summary score is a sum of all the five global items (items: 8, 13, 17, 22, 25), the minimum score is 0 and the maximum score is 25. SANS global summary scores were recorded in Belfast, Santander, and West London. The SANS composite score was recorded in Istanbul. The SANS item ‘inappropriate affect’ is sometimes dropped from the composite total score because it does not correlate with the overall subscale score^71^. This item was not recorded in both Belfast and West London. As individual items were not available, we decided to use SANS global summary scores. The method reported in Van Erp, Preda ^72^ was used to covert SANS composite total scores for participants from Istanbul into SANS global summary scores. The following equation was used: SANS global summary score = 1.0863+(0.2943*SANS composite total scores). The score for one individual was 25.8075, this was rounded down to 25.

### Scale for the Assessment of Positive Symptoms (SAPS)

SAPS scores were recorded in Belfast, Istanbul, Santander, and West London. The SAPS is used to rate positive symptoms in the following four domains: hallucinations, delusions, bizarre behaviour, and thought disorder^73^. Within each domain there are a number of individual items and one global item. Each item is rated on a 0-5 Likert scale (0 equates ‘absent’ and 5 equates to ‘severe’). The SAPS composite total is a sum of all SAPS items apart from the global items (items: 1-6, 8-19, 21-24, and 26-33). The SAPS global summary score is a sum of all the five global items (items: 7, 20, 25, 34). SANS composite total and global summary scores were recorded in Belfast, Istanbul, Santander, and West London. In line with the SANS scores available, only SANS global summary scores were used.

### Tobacco

Tobacco use (yes vs. no) was recorded at baseline in Belfast, Bologna, GAP, Santander, and West London. From this information, we created a binary variable: non-smoker vs. smoker.

### Years in education

Number of years in education was recorded at baseline for Belfast, Bologna, GAP, Istanbul, Oslo, Paris, Prague, and West London.

# Supplementary Methods

## Data Preparation

We began with N=2449 (TR N=329, 16%) participants from 12 cohorts. When clozapine alone was used as a definition of TR, there were N=2219 (TR N=244, 11%) participants. Descriptive statistics for the 26 potential predictors are available on request.

We removed cohorts where the number of TR cases was less than five (Oslo and Sao Paulo). This left a total sample size of N=2216 participants (TR=385; 17%).

Then we removed variables where more than 80% of the values were missing (we removed: family history of schizophrenia, family history of mental health disorders, supported accommodation, PANSS negative subscale, PANSS general psychopathology subscale, and PANSS total scores; Supplementary Table 1). No further variables became eligible for removal after dropping Oslo and Sao Paulo.

Next, we checked for multicollinearity and removed one variable from every pair of continuous or binary variables with a Pearson’s correlation greater than 0.8 (we removed: age at baseline, lifetime relationship; Supplementary Table 2). When correlating a dichotomous and continuous variable a point-biserial correlation is required, while Phi is required to correlate two dichotomous variables. Point-biserial correlations and Phi correlations are special cases of the Pearson correlation and therefore the same as computing the Pearson correlation. No further variables became eligible for removal after dropping Oslo and Sao Paulo.

We did not check the correlations between the three multi-level categorical variables (living situation, ethnicity and highest education qualification). However, given the suspected relationship between education years and highest education qualification (which we confirmed with a one-way ANOVA, F = 212, p < .001) we also removed highest education qualification.

Next, we checked the number of events/non-events per category for categorical variables. There were at least 10 TR cases or 10 non-TR controls per level for all the categorical variables, so none were removed.

For categorical variables the level with the largest number of participants was used as the reference level to improve avoid model estimations.

The final sample consisted of N=2216 (TR N=385, 17%) participants from 10 cohorts (Table 1). When clozapine alone was used as a definition of TR, there were N=1986 (TR N=238, 12%) participants. Descriptive statistics are presented in Table 2 for the 19 predictors retained.

## Explanatory Model

Multilevel MICE could not be applied to our data due to the low number of TR cases and the uneven distribution of observed data across cohorts. We compared MICE and multilevel MICE on a subsample of the data (consisting of a subsample of cohorts and variables) that fit the distributional assumptions and found no difference in log odds and standard error (data not shown, available on request).

## Prediction Model

No predictors had near zero variance. We would have removed any predictors which did have near zero variance.

Previous work by D.A. found that random forests imputation outperformed mice when paired with a LASSO regression (Agbedjro, 2018), plus, lasso regression counterbalances the known bias, that occurs due to overfitting (optimism), after random forests imputation^74^.

In the internal validation process, at each resampling step, we used the Lasso coefficients estimated in the training set in order to predict the probabilities of TR for each individual in the test set and get the test performances. The predicted probability for individual ‘i’ was computed according to the logistic regression formula:

p_i = exp(linear predictor_i)/(1+exp(linear predictor_i))

# Supplementary Results

## Explanatory Model

### Missing data imputation

Multiple imputation using chained equations performed well. All imputed values were within the range of observed values. For continuous variables, the distributions of imputed values were similar to the distributions of observed values (Figure 1). We checked convergence by examining trace lines, which we ideally expected to intermingle and be free of any trends at the later iterations (Figure 2). We checked the first five (out of the 100) imputed datasets by plotting (i) the jackknife deviance residuals against the fitted values, (ii) a QQ plot of the standardized deviance residuals, (iii) the Cook statistics against the standardized leverage, and (iv) the Cook statistic against case number (plots not shown). The residuals for the first five imputed datasets are available on request.

## Prediction Model

### Missing data imputation

Multiple imputation using random forests performed moderately well. The normalized root mean squared error (NRMSE) for the continuous variables was 52.25% and the proportion falsely classified (PFC) for the categorical variables was 19.15%.

### Lambda and the optimal cut-off point for the predicted probability (i.e., “decision thresholds”)

The lambda for the 1SE model was 0.014850.

Figure 3 shows how the AUC falls as lambdas increases. As can be seen below, the model with a 50% cut-off classed all participants as NTR and in doing so had an apparent accuracy of 82.63%. Therefore, we used a cut-off which maximised the sum of sensitivity and specificity, referred to as the ‘best’ threshold. The best threshold to discriminate between TR and NTR cases using the 1SE model was 20.21% which had an apparent accuracy of 71.30% (Figure 4).

### Performance measures

The apparent AUC ranged between 0.59 and 0.77 across the cohorts (Supplementary Table 7). The very high beta for the Prague sample are the result of some resampling models returning extreme negative performance, possibly due to very small prevalence of the outcome in those particular folds. The ‘mean’ as a measure of average takes the outliers into account.

Seven predictors were selected. The AUC for the apparent model was 0.65 but 0.59 after applying the optimism correction. See for Supplementary Table 8 other performance measures.

When clozapine was used as the definition of TR the dataset consisted of N=1986 participants, of which 238 (12%) were TR. The NRMSE for the continuous variables was 53.64% and the PFC for the categorical variables was 16.71%. The lambda for the 1SE model was 0.011768. The best threshold to discriminate between TR and NTR cases using the 1SE model was 12.45%. Seven predictors were selected. Performance measures are shown in Supplementary Table 9.

When only participants who had a diagnosis of schizophrenia at a follow up visit were used the dataset consisted of N=580 participants, of which 110 (19%) were TR. The NRMSE for the continuous variables was 76.87% and the PFC for the categorical variables was 12.24%. The lambda for the 1SE model was 0.02848. The best threshold to discriminate between TR and NTR cases using the 1SE model was 19.08%. Four predictors were selected. Performance measures are shown Supplementary Table 10.

### Equation

Equation 1. The equation to predict TR using 1SE LASSO logistic regression model with recalibrated coefficients.

$\mathrm{logit}\left( \hat{p} \right)$ provides the probability of being TR for each new observation. If the probability is over 20.21%, then the model would classify the observation as TR. If a patient’s probability of being TR is 90%, a clinician could be very certain that they are TR and will not respond to conventional antipsychotic medication. If a patient’s probability of being TR is 22%, the model would classify them as TR but a clinician may decide that treatment as usual is more appropriate than any alternative intervention.

$$\mathrm{logit}\left( \hat{p} \right)=\log\left( \frac{\hat{p}}{1-\hat{p}} \right)= -0.76 + -0.03\times age of onset \left( \mathrm{years} \right) -0.09\times gender \left( \mathrm{female} \right)+ 0.04 \times BMI -0.37\times current relationship \left( \mathrm{yes} \right) -0.06\times education \left( \mathrm{years} \right) -0.48\times alcohol \left( \mathrm{no} \right) -0.002\times PANSS positive subscale score$$

There is a script on how to produce a nomogram plot using the prediction model at: https://github.com/sophiesmart/stratagprediction. All analysis scripts can also be found in this repository.

# References

1. World Health Organization. The icd-10 classification of mental and behavioural disorders: Clinical descriptions and diagnostic guidelines: Geneva: World Health Organization; 1992.

2. Morgan C, Dazzan P, Morgan K, et al. First episode psychosis and ethnicity: Initial findings from the aesop study. *World Psychiatry* 2006; **5**(1): 40-6.

3. Fearon P, Kirkbride JB, Morgan C, et al. Incidence of schizophrenia and other psychoses in ethnic minority groups: Results from the mrc aesop study. *Psychological Medicine* 2006; **36**(11): 1541-50.

4. Zimbron J, Stahl D, Hutchinson G, et al. Pre-morbid fertility in psychosis: Findings from the aesop first episode study. *Schizophrenia Research* 2014; **156**(2): 168-73.

5. Dean K, Fearon P, Morgan K, et al. Grey matter correlates of minor physical anomalies in the æsop first-episode psychosis study. *British Journal of Psychiatry* 2018; **189**(3): 221-8.

6. Dazzan P, Morgan KD, Orr K, et al. Different effects of typical and atypical antipsychotics on grey matter in first episode psychosis: The æsop study. *Neuropsychopharmacology* 2005; **30**: 765.

7. Kirkbride JB, Fearon P, Morgan C, et al. Heterogeneity in incidence rates of schizophrenia and other psychotic syndromes: Findings from the 3-center aesop study. *Archives of General Psychiatry* 2006; **63**(3): 250-8.

8. Demjaha A, Lappin JM, Stahl D, et al. Antipsychotic treatment resistance in first-episode psychosis: Prevalence, subtypes and predictors. *Psychological Medicine* 2017; **47**(11): 1981-9.

9. Morgan C, Lappin J, Heslin M, et al. Reappraising the long-term course and outcome of psychotic disorders: The aesop-10 study. *Psychological Medicine* 2014; **44**(13): 2713-26.

10. Revier CJ, Reininghaus U, Dutta R, et al. Ten-year outcomes of first-episode psychoses in the mrc æsop-10 study. *The Journal of Nervous and Mental Disease* 2015; **203**(5): 379-86.

11. Di Forti M, Morgan C, Dazzan P, et al. High-potency cannabis and the risk of psychosis. *British Journal of Psychiatry* 2009; **195**(6): 488-91.

12. Di Forti M, Marconi A, Carra E, et al. Proportion of patients in south london with first-episode psychosis attributable to use of high potency cannabis: A case-control study. *The Lancet Psychiatry* 2015; **2**(3): 233-8.

13. Lally J, Ajnakina O, Di Forti M, et al. Two distinct patterns of treatment resistance: Clinical predictors of treatment resistance in first-episode schizophrenia spectrum psychoses. *Psychological Medicine* 2016; **46**(15): 3231-40.

14. Ajnakina O, Lally J, Di Forti M, et al. Patterns of illness and care over the 5 years following onset of psychosis in different ethnic groups; the gap-5 study. *Social Psychiatry and Psychiatric Epidemiology* 2017; **52**(9): 1101-11.

15. Stewart R, Soremekun M, Perera G, et al. The south london and maudsley nhs foundation trust biomedical research centre (slam brc) case register: Development and descriptive data. *BMC Psychiatry* 2009; **9**: 51.

16. Sartorius N, Gulbinat W, Harrison G, Laska E, Siegel C. Long-term follow-up of schizophrenia in 16 countries. A description of the international study of schizophrenia conducted by the world health organization. *Social Psychiatry and Psychiatric Epidemiology* 1996; **31**(5): 249-58.

17. McGuffin P, Farmer A, Harvey I. A polydiagnostic application of operational criteria in studies of psychotic illness: Development and reliability of the opcrit system. *JAMA Psychiatry* 1991; **48**(8): 764-70.

18. Turkington A, Mulholland CC, Rushe TM, et al. Impact of persistent substance misuse on 1-year outcome in first-episode psychosis. *British Journal of Psychiatry* 2018; **195**(3): 242-8.

19. American Psychiatric Association. Diagnostic and statistical manual of mental disorders dsm-iv-tr fourth edition (text revision). 2000.

20. Casey P, Corvin A. The clinical impact of substance use in schizophrenia: A study in an irish population. *TSMJ* 2008; **9**: 14-7.

21. Jongsma HE, Gayer-Anderson C, Lasalvia A, et al. Treated incidence of psychotic disorders in the multinational eu-gei study. *JAMA Psychiatry* 2018; **75**(1): 36-46.

22. Tarricone I, Mimmi S, Paparelli A, et al. First-episode psychosis at the west bologna community mental health centre: Results of an 8-year prospective study. *Psychological Medicine* 2012; **42**(11): 2255-64.

23. Üçok A, Çıkrıkçılı U, Ergül C, et al. Correlates of clozapine use after a first episode of schizophrenia: Results from a long-term prospective study. *CNS Drugs* 2016; **30**(10): 997-1006.

24. Ucok A, Polat A, Cakir S, Genc A. One year outcome in first episode schizophrenia. Predictors of relapse. *European Archives of Psychiatry and Clinical Neuroscience* 2006; **256**(1): 37-43.

25. Ucok A, Polat A, Genc A, Cakir S, Turan N. Duration of untreated psychosis may predict acute treatment response in first-episode schizophrenia. *Journal of Psychiatric Research* 2004; **38**(2): 163-8.

26. Ucok A, Serbest S, Kandemir PE. Remission after first-episode schizophrenia: Results of a long-term follow-up. *Psychiatry Research* 2011; **189**(1): 33-7.

27. Yung A, Phillips L, McGorry P, Ward J, Donovan K, Thompson K. Comprehensive assessment of at-risk mental states (caarms). Melbourne, Australia: Personal Assessment and Crisis Evaluation Clinic, Department of Psychiatry, University of Melbourne; 2002.

28. Alameda L, Golay P, Baumann PS, et al. Mild depressive symptoms mediate the impact of childhood trauma on long-term functional outcome in early psychosis patients. *Schizophrenia Bulletin* 2017; **43**(5): 1027-35.

29. Golay P, Alameda L, Baumann P, et al. Duration of untreated psychosis: Impact of the definition of treatment onset on its predictive value over three years of treatment. *Journal of Psychiatric Research* 2016; **77**: 15-21.

30. Baumann PS, Crespi S, Marion-Veyron R, et al. Treatment and early intervention in psychosis program (tipp-lausanne): Implementation of an early intervention programme for psychosis in switzerland. *Early Intervention in Psychiatry* 2013; **7**(3): 322-8.

31. Choong E, Quteineh L, Cardinaux JR, et al. Influence of crtc1 polymorphisms on body mass index and fat mass in psychiatric patients and the general adult population. *JAMA Psychiatry* 2013; **70**(10): 1011-9.

32. Delacretaz A, Preisig M, Vandenberghe F, et al. Influence of mchr2 and mchr2-as1 genetic polymorphisms on body mass index in psychiatric patients and in population-based subjects with present or past atypical depression. *PloS One* 2015; **10**(10): e0139155.

33. Quteineh L, Vandenberghe F, Saigi Morgui N, et al. Impact of hsd11b1 polymorphisms on bmi and components of the metabolic syndrome in patients receiving psychotropic treatments. *Pharmacogenetics and Genomics* 2015; **25**(5): 246-58.

34. Vandenberghe F, Gholam-Rezaee M, Saigi-Morgui N, et al. Importance of early weight changes to predict long-term weight gain during psychotropic drug treatment. *The Journal of Clinical Psychiatry* 2015; **76**(11): e1417-23.

35. Choong E, Solida A, Lechaire C, Conus P, Eap CB. Follow-up of the metabolic syndrome induced by atypical antipsychotics: Recommendations and pharmacogenetics perspectives. *Revue Médicale Suisse* 2008; **4**(171): 1994-6,8-9.

36. Athanasiu L, Mattingsdal M, Kahler AK, et al. Gene variants associated with schizophrenia in a norwegian genome-wide study are replicated in a large european cohort. *Journal of Psychiatric Research* 2010; **44**(12): 748-53.

37. Faerden A, Nesvag R, Barrett EA, et al. Assessing apathy: The use of the apathy evaluation scale in first episode psychosis. *European Psychiatry* 2008; **23**(1): 33-9.

38. Lyngstad SH, Gardsjord ES, Simonsen C, et al. Consequences of persistent depression and apathy in first-episode psychosis — a one-year follow-up study. *Comprehensive Psychiatry* 2018; **86**: 60-6.

39. Lange EH, Nesvåg R, Ringen PA, et al. One year follow-up of alcohol and illicit substance use in first-episode psychosis: Does gender matter? *Comprehensive Psychiatry* 2014; **55**(2): 274-82.

40. Faerden A, Barrett EA, Nesvåg R, et al. Apathy, poor verbal memory and male gender predict lower psychosocial functioning one year after the first treatment of psychosis. *Psychiatry Research* 2013; **210**(1): 55-61.

41. Kolenic M, Franke K, Hlinka J, et al. Obesity, dyslipidemia and brain age in first-episode psychosis. *Journal of Psychiatric Research* 2018; **99**: 151-8.

42. Spaniel F, Tintera J, Rydlo J, et al. Altered neural correlate of the self-agency experience in first-episode schizophrenia-spectrum patients: An fmri study. *Schizophrenia Bulletin* 2016; **42**(4): 916-25.

43. Mikolas P, Melicher T, Skoch A, et al. Connectivity of the anterior insula differentiates participants with first-episode schizophrenia spectrum disorders from controls: A machine-learning study. *Psychological Medicine* 2016; **46**(13): 2695-704.

44. Melicher T, Horacek J, Hlinka J, et al. White matter changes in first episode psychosis and their relation to the size of sample studied: A dti study. *Schizophrenia Research* 2015; **162**(1-3): 22-8.

45. Ayesa-Arriola R, Teran JMP, Morinigo JDL, et al. The dynamic relationship between insight and suicidal behavior in first episode psychosis patients over 3-year follow-up. *European Neuropsychopharmacology* 2018.

46. Setien-Suero E, Martinez-Garcia O, de la Foz VO, et al. Age of onset of cannabis use and cognitive function in first-episode non-affective psychosis patients: Outcome at three-year follow-up. *Schizophrenia Research* 2018.

47. Pelayo-Teran JM, Perez-Iglesias R, Ramirez-Bonilla M, et al. Epidemiological factors associated with treated incidence of first-episode non-affective psychosis in cantabria: Insights from the clinical programme on early phases of psychosis. *Early Intervention in Psychiatry* 2008; **2**(3): 178-87.

48. Crespo-Facorro B, Pelayo-Teran JM, Perez-Iglesias R, et al. Predictors of acute treatment response in patients with a first episode of non-affective psychosis: Sociodemographics, premorbid and clinical variables. *Journal of Psychiatric Research* 2007; **41**(8): 659-66.

49. Menezes PR, Scazufca M, Busatto GF, Coutinho LM, McGuire PK, Murray RM. Incidence of first-contact psychosis in sao paulo, brazil. *British Journal of Psychiatry* 2007; **191**(S51): s102-s6.

50. Ayres AM, Busatto GF, Menezes PR, et al. Cognitive deficits in first-episode psychosis: A population-based study in sao paulo, brazil. *Schizophrenia Research* 2007; **90**(1-3): 338-43.

51. Martinho Jr E, Michelon L, Ayres AM, et al. Bdnf gene polymorphism, cognition and symptom severity in a brazilian population-based sample of first-episode psychosis subjects. *Brazilian Journal of Psychiatry* 2012; **34**: 219-32.

52. Schaufelberger M, Lappin J, Duran F, et al. Lack of progression of brain abnormalities in first-episode psychosis: A longitudinal magnetic resonance imaging study. *Psychological Medicine* 2011; **41**(8): 1677-89.

53. Minatogawa-Chang TM, Schaufelberger MS, Ayres AM, et al. Cognitive performance is related to cortical grey matter volumes in early stages of schizophrenia: A population-based study of first-episode psychosis. *Schizophrenia Research* 2009; **113**(2-3): 200-9.

54. Barnes TRE, Leeson VC, Mutsatsa SH, Watt HC, Hutton SB, Joyce EM. Duration of untreated psychosis and social function: 1-year follow-up study of first-episode schizophrenia. *British Journal of Psychiatry* 2008; **193**(3): 203-9.

55. Gutierrez-Galve L, Chu EM, Leeson VC, et al. A longitudinal study of cortical changes and their cognitive correlates in patients followed up after first-episode psychosis. *Psychological Medicine* 2015; **45**(1): 205-16.

56. Gutierrez-Galve L, Wheeler-Kingshott CA, Altmann DR, et al. Changes in the frontotemporal cortex and cognitive correlates in first-episode psychosis. *Biological Psychiatry* 2010; **68**(1): 51-60.

57. Huddy VC, Clark L, Harrison I, et al. Reflection impulsivity and response inhibition in first-episode psychosis: Relationship to cannabis use. *Psychological Medicine* 2013; **43**(10): 2097-107.

58. Huddy VC, Hodgson TL, Kapasi M, et al. Gaze strategies during planning in first-episode psychosis. *Journal of Abnormal Psychology* 2007; **116**(3): 589-98.

59. Leeson VC, Barnes TRE, Hutton SB, Ron MA, Joyce EM. Iq as a predictor of functional outcome in schizophrenia: A longitudinal, four-year study of first-episode psychosis. *Schizophrenia Research* 2009; **107**(1): 55-60.

60. Leeson VC, Harrison I, Ron MA, Barnes TRE, Joyce EM. The effect of cannabis use and cognitive reserve on age at onset and psychosis outcomes in first-episode schizophrenia. *Schizophrenia Bulletin* 2012; **38**(4): 873-80.

61. Leeson VC, Robbins TW, Matheson E, et al. Discrimination learning, reversal, and set-shifting in first-episode schizophrenia: Stability over six years and specific associations with medication type and disorganization syndrome. *Biological Psychiatry* 2009; **66**(6): 586-93.

62. Leeson VC, Sharma P, Harrison M, Ron MA, Barnes TRE, Joyce EM. Iq trajectory, cognitive reserve, and clinical outcome following a first episode of psychosis: A 3-year longitudinal study. *Schizophrenia Bulletin* 2011; **37**(4): 768-77.

63. Howes OD, McCutcheon R, Agid O, et al. Treatment-resistant schizophrenia: Treatment response and resistance in psychosis (trrip) working group consensus guidelines on diagnosis and terminology. *The American Journal of Psychiatry* 2017; **174**(3): 216-29.

64. Drake RE, Osher FC, Noordsy DL, Hurlbut SC, Teague GB, Beaudett MS. Diagnosis of alcohol use disorders in schizophrenia. *Schizophrenia Bulletin* 1990; **16**(1): 57-67.

65. Overall JE, Gorham DR. The brief psychiatric rating scale. *Psychological Reports* 1962; **10**(3): 799-812.

66. Maxwell ME. Family interview for genetic studies (figs): A manual for figs. Clinical Neurogenetics Branch, Intramural Research Program, National Institute of Mental Health: Bethesda, MD; 1992.

67. Castle D, Jablensky A, McGrath J, et al. The diagnostic interview for psychoses (dip): Development, reliability and applications. *Psychological Medicine* 2006; **36**(1): 69-80.

68. Kay SR, Fiszbein A, Opler LA. The positive and negative syndrome scale (panss) for schizophrenia. *Schizophrenia Bulletin* 1987; **13**(2): 261-76.

69. Andreasen NC. The scale for the assessment of negative symptoms (sans). Iowa City, Iowa: The University of Iowa; 1983.

70. Andreasen NC. The scale for the assessment of negative symptoms (sans): Conceptual and theoretical foundations. *British Journal of Psychiatry* 1989; **155**(S7): 49-52.

71. Andreasen NC. Negative symptoms in schizophrenia: Definition and reliability. *Archives Of General Psychiatry* 1982; **39**(7): 784-8.

72. Van Erp TG, Preda A, Nguyen D, et al. Converting positive and negative symptom scores between panss and saps/sans. *Schizophrenia Research* 2014; **152**(1): 289-94.

73. Andreasen NC. The scale for the assessment of positive symptoms (saps). Iowa City, Iowa: The University of Iowa; 1984.

74. Lu F, Petkova E. A comparative study of variable selection methods in the context of developing psychiatric screening instruments. *Statistics in Medicine* 2014; **33**(3): 401-21.

# Supplementary Tables & Figures

**Supplementary Table 1.** Proportion of missing data, stratified by cohort.

**Supplementary Table 2.** Correlation matrix.

**Supplementary Table 3.** Results of the univariable logistic regressions (explanatory models).

**Supplementary Table 4.** Results of the multivariable logistic regression (explanatory model), including covariates.

**Supplementary Table 5.** Results of the multivariable LASSO regressions (prediction models), including both apparent regression coefficients and recalibrated regression coefficients.

**Supplementary Table 6.** Criteria used to define treatment resistance stratified by cohort.

**Supplementary Table 7.** Average apparent performance measures for each cohort after repeated 5-fold validation (5 folds, 50 repeats).

**Supplementary Table 8.** Apparent and corrected performance measures for the 1SE logistic LASSO regression, when using both a 50% threshold and the ‘best’ threshold.

**Supplementary Table 9.** Clozapine Model: Apparent and corrected performance measures for the 1SE logistic LASSO regression, when using both a 50% threshold and the ‘best’ threshold.

**Supplementary Table 10.** Schizophrenia Model: Apparent and corrected performance measures for the 1SE logistic LASSO regression, when using both a 50% threshold and the ‘best’ threshold.

**Figure 1.** Multiple imputation using chained equations (MICE) density plots for imputed continuous variables.

**Figure 2.** Multiple imputation using chained equations (MICE) chain plots showing trace lines for imputed values.

**Figure 3.** Plot showing lambda plotted against the receiver operating characteristic curve.

**Figure 4.** Plot showing the best threshold and apparent AUC for the 1SE logistic LASSO regression.

| Supplementary Table 1. Proportion of missing data, stratified by cohort. | | | | | | | | | | | | | |
| --- | --- | --- | --- | --- | --- | --- | --- | --- | --- | --- | --- | --- | --- |
| **All Predictors** | **All** | **Santander** | **AESOP London** | **Belfast** | **Bologna** | **GAP London** | **Istanbul** | **Lausanne** | **Oslo** | **Paris** | **Prague** | **Sao Paulo** | **UCL London** |
| Cohort | 0.00 | 0.00 | 0.00 | 0.00 | 0.00 | 0.00 | 0.00 | 0.00 | 0.00 | 0.00 | 0.00 | 0.00 | 0.00 |
| Clozapine | 9.39 | 0.00 | 72.38 | 0.00 | 0.00 | 8.01 | 0.00 | 0.00 | 0.00 | 0.00 | 0.00 | 0.00 | 0.00 |
| Treatment Resistant Status | 0.00 | 0.00 | 0.00 | 0.00 | 0.00 | 0.00 | 0.00 | 0.00 | 0.00 | 0.00 | 0.00 | 0.00 | 0.00 |
| Length of follow up | 2.65 | 0.00 | 0.00 | 0.00 | 0.00 | 0.00 | 0.00 | 0.00 | 0.00 | 0.00 | 0.00 | 100.00 | 0.00 |
| Age at onset | 15.43 | 2.74 | 0.70 | 27.45 | 0.00 | 2.44 | 0.74 | 33.57 | 0.00 | 3.13 | 100.00 | 100.00 | 3.44 |
| Duration of untreated psychosis | 27.52 | 2.74 | 8.39 | 34.64 | 100.00 | 100.00 | 1.48 | 33.21 | 0.00 | 6.25 | 56.43 | 100.00 | 1.59 |
| Gender | 4.94 | 0.21 | 0.00 | 0.00 | 0.00 | 0.00 | 0.00 | 0.00 | 0.00 | 0.00 | 55.71 | 3.08 | 10.58 |
| BMI | 64.15 | 1.47 | 100.00 | 47.71 | 100.00 | 42.16 | 100.00 | 100.00 | 2.38 | 100.00 | 100.00 | 100.00 | 100.00 |
| Current relationship | 30.42 | 0.63 | 7.34 | 6.54 | 18.00 | 29.27 | 2.22 | 33.21 | 0.00 | 3.13 | 55.71 | 100.00 | 100.00 |
| Living situation | 52.47 | 0.63 | 7.34 | 72.55 | 18.00 | 29.62 | 100.00 | 100.00 | 15.48 | 100.00 | 100.00 | 100.00 | 100.00 |
| Employment | 72.68 | 100.00 | 100.00 | 13.73 | 18.00 | 31.01 | 2.22 | 100.00 | 19.64 | 3.13 | 100.00 | 100.00 | 100.00 |
| Education (years) | 66.97 | 100.00 | 100.00 | 37.91 | 18.00 | 59.93 | 0.00 | 100.00 | 0.00 | 12.50 | 55.71 | 100.00 | 56.35 |
| Cannabis | 47.16 | 0.21 | 100.00 | 28.10 | 20.00 | 42.86 | 100.00 | 36.43 | 1.19 | 100.00 | 100.00 | 3.08 | 73.81 |
| Tobacco | 57.94 | 0.42 | 100.00 | 1.31 | 20.00 | 48.43 | 100.00 | 100.00 | 100.00 | 100.00 | 100.00 | 3.08 | 58.99 |
| Alcohol | 55.94 | 0.84 | 100.00 | 28.10 | 100.00 | 100.00 | 100.00 | 36.43 | 1.19 | 100.00 | 100.00 | 100.00 | 59.26 |
| PANSS positive subscale | 72.40 | 100.00 | 100.00 | 30.72 | 100.00 | 39.72 | 100.00 | 62.50 | 0.00 | 100.00 | 56.43 | 3.08 | 100.00 |
| SAPS | 58.31 | 0.21 | 100.00 | 74.51 | 100.00 | 100.00 | 2.96 | 100.00 | 100.00 | 100.00 | 100.00 | 100.00 | 0.26 |
| SANS | 59.33 | 0.84 | 100.00 | 75.16 | 100.00 | 100.00 | 17.78 | 100.00 | 100.00 | 100.00 | 100.00 | 100.00 | 0.53 |
| BPRS | 75.30 | 0.84 | 100.00 | 100.00 | 100.00 | 100.00 | 0.74 | 100.00 | 100.00 | 100.00 | 100.00 | 100.00 | 100.00 |
| GAF | 73.17 | 100.00 | 100.00 | 71.24 | 100.00 | 59.58 | 12.59 | 45.00 | 13.10 | 46.88 | 55.71 | 100.00 | 100.00 |
| Ethnicity | 31.20 | 0.63 | 2.80 | 82.35 | 0.00 | 0.00 | 0.00 | 3.57 | 1.19 | 100.00 | 100.00 | 100.00 | 100.00 |
| Schizophrenia diagnosis | 0.00 | 0.00 | 0.00 | 0.00 | 0.00 | 0.00 | 0.00 | 0.00 | 0.00 | 0.00 | 0.00 | 0.00 | 0.00 |
| Abbreviations: BMI, Body Mass Index; BPRS, Brief Psychiatric Rating Scale; GAF, Global Assessment of Functioning; PANSS, Positive and Negative Syndrome Scale; SANS, Scale for the Assessment of Negative Symptoms; SAPS, Scale for the Assessment of Positive Symptoms. | | | | | | | | | | | | | |

| Supplementary Table 2. Correlation matrix. | | | | | | | | | | | | | | | |
| --- | --- | --- | --- | --- | --- | --- | --- | --- | --- | --- | --- | --- | --- | --- | --- |
|  | **Gender (Male; %)** | **Current relationship (Yes; %)** | **Employment (Employed; %)** | **Cannabis (Yes; %)** | **Tobacco (Yes; %)** | **Alcohol (Yes; %)** | **Age at onset** | **Duration of untreated psychosis** | **BMI** | **Education (years)** | **PANSS positive subscale** | **SAPS** | **SANS** | **BPRS** | **GAF** |
| Gender (Male; %) | 1.00 | 0.19 | 0.00 | -0.26 | 0.14 | 0.18 | 0.20 | -0.03 | -0.13 | 0.11 | -0.08 | -0.04 | -0.10 | -0.03 | 0.10 |
| Current relationship (Yes; %) | 0.19 | 1.00 | -0.02 | -0.17 | 0.11 | 0.12 | 0.28 | 0.07 | 0.10 | 0.03 | -0.10 | -0.05 | -0.12 | -0.05 | 0.09 |
| Employment (Employed; %) | 0.00 | -0.02 | 1.00 | -0.03 | 0.05 | -0.05 | -0.12 | -0.01 | -0.07 | 0.23 | -0.14 | -0.08 | -0.18 | -0.07 | 0.19 |
| Cannabis (Yes; %) | -0.26 | -0.17 | -0.03 | 1.00 | -0.39 | -0.23 | -0.27 | 0.00 | -0.12 | -0.02 | 0.18 | 0.05 | 0.10 | 0.05 | -0.10 |
| Tobacco (Yes; %) | 0.14 | 0.11 | 0.05 | -0.39 | 1.00 | 0.27 | 0.10 | 0.05 | 0.07 | 0.04 | -0.03 | -0.05 | -0.05 | -0.03 | 0.38 |
| Alcohol (Yes; %) | 0.18 | 0.12 | -0.05 | -0.23 | 0.27 | 1.00 | 0.21 | -0.06 | 0.00 | -0.09 | 0.07 | -0.11 | 0.06 | -0.01 | -0.09 |
| Age at onset | 0.20 | 0.28 | -0.12 | -0.27 | 0.10 | 0.21 | 1.00 | -0.08 | 0.14 | 0.13 | -0.01 | 0.05 | -0.23 | -0.06 | 0.02 |
| Duration of untreated psychosis | -0.03 | 0.07 | -0.01 | 0.00 | 0.05 | -0.06 | -0.08 | 1.00 | 0.09 | -0.09 | 0.12 | -0.08 | 0.17 | -0.03 | -0.21 |
| BMI | -0.13 | 0.10 | -0.07 | -0.12 | 0.07 | 0.00 | 0.14 | 0.09 | 1.00 | 0.01 | -0.09 | -0.02 | -0.04 | -0.05 | 0.02 |
| Education | 0.11 | 0.03 | 0.23 | -0.02 | 0.04 | -0.09 | 0.13 | -0.09 | 0.01 | 1.00 | -0.07 | -0.07 | -0.23 | -0.18 | 0.21 |
| PANSS positive subscale | -0.08 | -0.10 | -0.14 | 0.18 | -0.03 | 0.07 | -0.01 | 0.12 | -0.09 | -0.07 | 1.00 | NA | NA | NA | -0.39 |
| SAPS | -0.04 | -0.05 | -0.08 | 0.05 | -0.05 | -0.11 | 0.05 | -0.08 | -0.02 | -0.07 | NA | 1.00 | 0.10 | 0.61 | -0.27 |
| SANS | -0.10 | -0.12 | -0.18 | 0.10 | -0.05 | 0.06 | -0.23 | 0.17 | -0.04 | -0.23 | NA | 0.10 | 1.00 | 0.31 | -0.08 |
| BPRS | -0.03 | -0.05 | -0.07 | 0.05 | -0.03 | -0.01 | -0.06 | -0.03 | -0.05 | -0.18 | NA | 0.61 | 0.31 | 1.00 | -0.26 |
| GAF | 0.10 | 0.09 | 0.19 | -0.10 | 0.38 | -0.09 | 0.02 | -0.21 | 0.02 | 0.21 | -0.39 | -0.27 | -0.08 | -0.26 | 1.00 |
| Abbreviations: BMI, Body Mass Index; BPRS, Brief Psychiatric Rating Scale; GAF, Global Assessment of Functioning; PANSS, Positive and Negative Syndrome Scale; SANS, Scale for the Assessment of Negative Symptoms; SAPS, Scale for the Assessment of Positive Symptoms. | | | | | | | | | | | | | | | |

| Supplementary Table 3. Results of the univariable logistic regressions (explanatory models). | | | | | | | | | |
| --- | --- | --- | --- | --- | --- | --- | --- | --- | --- |
|  | **Estimate** | **95% CI** | | **Std. Error** | **Odds Ratio** | **95% CI** | | **P-Value** |  |
| Length of follow up | 0.11 | 0.08 | 0.14 | 0.02 | 1.12 | 1.09 | 1.15 | 0.000 | *** |
| AESOP London | 0.83 | 0.45 | 1.20 | 0.19 | 2.28 | 1.91 | 2.65 | 0.000 | *** |
| Belfast | -0.38 | -0.97 | 0.22 | 0.30 | 0.69 | 0.09 | 1.28 | 0.213 |  |
| Bologna | 0.03 | -0.81 | 0.87 | 0.43 | 1.03 | 0.19 | 1.87 | 0.951 |  |
| GAP London | 0.73 | 0.35 | 1.10 | 0.19 | 2.07 | 1.70 | 2.45 | 0.000 | *** |
| Istanbul | 0.87 | 0.41 | 1.33 | 0.23 | 2.38 | 1.92 | 2.84 | 0.000 | *** |
| Lausanne | -0.28 | -0.74 | 0.18 | 0.23 | 0.76 | 0.30 | 1.22 | 0.236 |  |
| Paris | 0.38 | -0.55 | 1.30 | 0.47 | 1.46 | 0.53 | 2.38 | 0.427 |  |
| Prague | 1.67 | 1.25 | 2.09 | 0.22 | 5.31 | 4.89 | 5.73 | 0.000 | *** |
| UCL London | -1.42 | -2.01 | -0.82 | 0.30 | 0.24 | -0.35 | 0.84 | 0.000 | *** |
| Age at onset (years) | -0.03 | -0.05 | -0.02 | 0.01 | 0.97 | 0.95 | 0.98 | 0.000 | *** |
| Duration of untreated psychosis (days) | 0.00 | 0.00 | 0.00 | 0.00 | 1.00 | 1.00 | 1.00 | 0.368 |  |
| Gender = female | -0.30 | -0.55 | -0.05 | 0.13 | 0.74 | 0.49 | 0.99 | 0.020 | * |
| BMI | 0.04 | 0.01 | 0.08 | 0.02 | 1.04 | 1.01 | 1.08 | 0.019 | * |
| Current relationship = yes | -0.75 | -1.16 | -0.35 | 0.21 | 0.47 | 0.07 | 0.87 | 0.000 | *** |
| Living situation = alone | 0.11 | -0.22 | 0.44 | 0.17 | 1.11 | 0.79 | 1.44 | 0.516 |  |
| Living situation = with others | 0.41 | -0.02 | 0.84 | 0.22 | 1.51 | 1.08 | 1.94 | 0.063 |  |
| Employment = employed | -0.10 | -0.56 | 0.35 | 0.23 | 0.90 | 0.45 | 1.35 | 0.652 |  |
| Education (years) | -0.08 | -0.14 | -0.02 | 0.03 | 0.92 | 0.86 | 0.98 | 0.012 | * |
| Cannabis = yes | -0.07 | -0.39 | 0.26 | 0.17 | 0.94 | 0.61 | 1.26 | 0.687 |  |
| Tobacco = no | -0.22 | -0.64 | 0.20 | 0.21 | 0.80 | 0.38 | 1.22 | 0.302 |  |
| Alcohol = no | -0.11 | -0.50 | 0.28 | 0.20 | 0.90 | 0.51 | 1.29 | 0.582 |  |
| PANSS positive subscale | 0.03 | -0.01 | 0.07 | 0.02 | 1.03 | 0.99 | 1.07 | 0.200 |  |
| SAPS | 0.05 | 0.00 | 0.10 | 0.03 | 1.05 | 1.00 | 1.11 | 0.039 | * |
| SANS | 0.01 | -0.02 | 0.04 | 0.02 | 1.01 | 0.98 | 1.04 | 0.379 |  |
| BPRS | 0.01 | -0.01 | 0.02 | 0.01 | 1.01 | 0.99 | 1.02 | 0.403 |  |
| GAF | -0.02 | -0.03 | 0.00 | 0.01 | 0.98 | 0.97 | 1.00 | 0.019 | * |
| Ethnicity = African | 0.40 | 0.11 | 0.70 | 0.15 | 1.50 | 1.20 | 1.80 | 0.008 | ** |
| Ethnicity = Asian/Mixed/Other | 0.02 | -0.44 | 0.48 | 0.23 | 1.02 | 0.56 | 1.48 | 0.930 |  |
| NB: The reference category for living situation was ‘living with family’ and for ethnicity the reference category was ‘European’.  Abbreviations: BMI, Body Mass Index; BPRS, Brief Psychiatric Rating Scale; GAF, Global Assessment of Functioning; PANSS, Positive and Negative Syndrome Scale; SANS, Scale for the Assessment of Negative Symptoms; SAPS, Scale for the Assessment of Positive Symptoms. | | | | | | | | | |

| Supplementary Table 4. Results of the multivariable logistic regression (explanatory model), including covariates. | | | | | | | | | |
| --- | --- | --- | --- | --- | --- | --- | --- | --- | --- |
|  | **Estimate** | **95% CI** | | **Std. Error** | **Odds Ratio** | **95% CI** | | **P-Value** |  |
| (Intercept) | -0.29 | -3.34 | 2.77 | 1.56 | 0.75 | -2.30 | 3.81 | 0.855 |  |
| Length of follow up | 0.20 | 0.17 | 0.27 | 0.03 | 1.22 | 1.16 | 1.29 | 0.000 | *** |
| AESOP London | -0.54 | -0.89 | 0.10 | 0.33 | 0.58 | -0.06 | 1.22 | 0.096 |  |
| Belfast | -0.56 | -1.03 | 0.31 | 0.45 | 0.57 | -0.30 | 1.45 | 0.209 |  |
| Bologna | 0.27 | -0.25 | 1.24 | 0.49 | 1.32 | 0.35 | 2.28 | 0.576 |  |
| GAP London | 0.27 | -0.05 | 0.88 | 0.31 | 1.32 | 0.71 | 1.92 | 0.374 |  |
| Istanbul | 0.01 | -0.34 | 0.67 | 0.33 | 1.01 | 0.36 | 1.67 | 0.966 |  |
| Lausanne | -0.46 | -0.78 | 0.14 | 0.30 | 0.63 | 0.04 | 1.23 | 0.132 |  |
| Paris | -0.12 | -0.69 | 0.93 | 0.54 | 0.89 | -0.16 | 1.94 | 0.826 |  |
| Prague | 2.99 | 2.62 | 3.68 | 0.35 | 19.90 | 19.21 | 20.59 | 0.000 | *** |
| UCL London | -1.64 | -2.03 | -0.93 | 0.37 | 0.19 | -0.52 | 0.91 | 0.000 | *** |
| Age at onset (years) | -0.04 | -0.05 | -0.02 | 0.01 | 0.96 | 0.94 | 0.98 | 0.000 | *** |
| Duration of untreated psychosis (days) | 0.00 | 0.00 | 0.00 | 0.00 | 1.00 | 1.00 | 1.00 | 0.821 |  |
| Gender = female | -0.12 | -0.32 | 0.24 | 0.18 | 0.89 | 0.53 | 1.25 | 0.518 |  |
| BMI | 0.05 | 0.02 | 0.11 | 0.03 | 1.05 | 0.99 | 1.11 | 0.089 |  |
| Current relationship = yes | -0.44 | -0.73 | 0.08 | 0.27 | 0.64 | 0.12 | 1.17 | 0.098 |  |
| Living situation = alone | -0.08 | -0.35 | 0.42 | 0.25 | 0.92 | 0.43 | 1.42 | 0.754 |  |
| Living situation = with others | 0.42 | 0.10 | 0.99 | 0.30 | 1.52 | 0.94 | 2.09 | 0.160 |  |
| Employment = employed | 0.03 | -0.30 | 0.63 | 0.31 | 1.03 | 0.43 | 1.63 | 0.922 |  |
| Education (years) | -0.14 | -0.18 | -0.06 | 0.04 | 0.87 | 0.79 | 0.95 | 0.001 | *** |
| Cannabis = yes | 0.01 | -0.30 | 0.57 | 0.29 | 1.01 | 0.45 | 1.57 | 0.981 |  |
| Tobacco = no | 0.07 | -0.32 | 0.78 | 0.37 | 1.07 | 0.35 | 1.79 | 0.858 |  |
| Alcohol = no | -0.14 | -0.52 | 0.55 | 0.35 | 0.87 | 0.17 | 1.56 | 0.686 |  |
| PANSS positive subscale | 0.03 | -0.01 | 0.12 | 0.04 | 1.04 | 0.95 | 1.12 | 0.435 |  |
| SAPS | 0.08 | 0.02 | 0.19 | 0.05 | 1.09 | 0.98 | 1.19 | 0.134 |  |
| SANS | 0.00 | -0.03 | 0.07 | 0.03 | 1.00 | 0.94 | 1.07 | 0.887 |  |
| BPRS | -0.02 | -0.04 | 0.02 | 0.02 | 0.98 | 0.95 | 1.02 | 0.297 |  |
| GAF | -0.02 | -0.04 | 0.00 | 0.01 | 0.98 | 0.95 | 1.00 | 0.059 |  |
| Ethnicity = African | 0.00 | -0.46 | 0.84 | 0.43 | 1.00 | 0.15 | 1.84 | 0.991 |  |
| Ethnicity = Asian/Mixed/Other | -0.16 | -0.52 | 0.49 | 0.33 | 0.85 | 0.19 | 1.51 | 0.630 |  |
| NB: The reference category for living situation was ‘living with family’ and for ethnicity the reference category was ‘European’.  Abbreviations: BMI, Body Mass Index; BPRS, Brief Psychiatric Rating Scale; GAF, Global Assessment of Functioning; PANSS, Positive and Negative Syndrome Scale; SANS, Scale for the Assessment of Negative Symptoms; SAPS, Scale for the Assessment of Positive Symptoms. | | | | | | | | | |

| Supplementary Table 5. Results of the multivariable LASSO regressions (prediction models), including both apparent regression coefficients and recalibrated regression coefficients. | | | | | | |
| --- | --- | --- | --- | --- | --- | --- |
|  | **Original Model**  **(N=2216)** | | **Clozapine-only Model**  **(N=1986)** | | **Schizophrenia-only Model**  **(N=580)** | |
|  | **Apparent** | **Recalibrated** | **Apparent** | **Recalibrated** | **Apparent** | **Recalibrated** |
| (Intercept) | -0.9779192 | -1.279978 | -1.38519315 | -1.921656 | -1.1011882 | -2.70145 |
| Age at onset (years) | -0.0196114 | -0.0264154 | -0.02872447 | -0.040122727 | -0.0084106 | -0.0206642 |
| Duration of untreated psychosis (days) | 0 | 0 | 0 | 0 | 0 | 0 |
| Gender = female | -0.065621 | -0.0883877 | 0 | 0 | -0.0526674 | -0.1294004 |
| BMI | 0.02574458 | 0.03467645 | 0 | 0 | 0 | 0 |
| Current relationship = yes | -0.2731482 | -0.3679146 | -0.04017068 | -0.056110946 | -0.3091839 | -0.7596446 |
| Living = alone | 0 | 0 | 0 | 0 | 0 | 0 |
| Living = with others | 0 | 0 | 0 | 0 | 0 | 0 |
| Employment = employed | 0 | 0 | -0.00871166 | -0.012168568 | -0.1446347 | -0.3553579 |
| Education (years) | -0.0413125 | -0.0556455 | 0 | 0 | 0 | 0 |
| Cannabis = yes | 0 | 0 | 0 | 0 | 0 | 0 |
| Tobacco = no | 0 | 0 | -0.17302977 | -0.241690338 | 0 | 0 |
| Alcohol = no | -0.3525363 | -0.4748458 | -0.08209876 | -0.11467666 | 0 | 0 |
| PANSS positive subscale | -0.0012722 | -0.0017135 | 0 | 0 | 0 | 0 |
| SAPS | 0 | 0 | 0 | 0 | 0 | 0 |
| SANS | 0 | 0 | 0.020400029 | 0.028495037 | 0 | 0 |
| BPRS | 0 | 0 | 0 | 0 | 0 | 0 |
| GAF | 0 | 0 | 0 | 0 | 0 | 0 |
| Ethnicity = African | 0 | 0 | 0.179558161 | 0.25080928 | 0 | 0 |
| Ethnicity = Asian/Mixed/Other | 0 | 0 | 0 | 0 | 0 | 0 |
| NB: The clozapine-only model was developed using a subsample of participants where TR was defined using only clozapine prescription. The schizophrenia-only model was developed using a subsample of participants with a diagnosis of schizophrenia (at the last known follow-up visit). The reference category for living situation was ‘living with family’ and for ethnicity the reference category was ‘European’.  Abbreviations: BMI, Body Mass Index; BPRS, Brief Psychiatric Rating Scale; GAF, Global Assessment of Functioning; PANSS, Positive and Negative Syndrome Scale; SANS, Scale for the Assessment of Negative Symptoms; SAPS, Scale for the Assessment of Positive Symptoms. | | | | | | |

| Supplementary Table 6. Criteria used to define treatment resistance stratified by cohort. | | | | | | | | |
| --- | --- | --- | --- | --- | --- | --- | --- | --- |
| **Criteria/**  **Guidelines** | **Diagnoses at baseline recruitment** | **Number of antipsychotic medications** | **Type of antipsychotic medications** | **Duration of antipsychotic trial** | **Dosage during trial** | **Clinical response** | **Functional response** | **Adherence** |
| AESOP (London, UK) | ICD-10 diagnosis of F10-F29 or F30-F33 | 2 | NS | ≥4 weeks | Daily dose of 400–600 mg of chlorpromazine equivalents | At least moderate severity on one or more positive symptoms as rated by SCAN (ICD-10) | NS | Recorded adherence to medication |
| NIFEPS & RGPI (Belfast, UK) | NIFEPS: OPCRIT diagnosis of first episode psychosis  RGPI: DSM-IV diagnosis of schizophrenia, schizophreniform disorder, schizoaffective disorder or bipolar affective disorder with psychosis | 2 | At least two different antipsychotics | ≥6 weeks | At least the mid-point of the licensed therapeutic range | At least moderate severity (a score of more than 70), as rated by the PANSS for at least 12 weeks | At least moderate functional impairment measured using the GAF scale (a score less than or equal to 51) | NS |
| EUGEI & BoFEP (Bologna, Italy) | EUGEI: ICD-10 diagnosis of F20-F33  BoFEP: ICD-10 diagnosis of F10–F29 or F30–F33 | 2 | At least two different antipsychotics | ≥6 weeks | At least the mid-point of the licensed therapeutic range | A rating of at least 70 on the PANSS | At least moderate functional impairment measured using the CGI scale | NS |
| Gap (London, UK)^1^ | ICD-10 diagnosis of F20-F29 or F30-F33 | 2 | NS | ≥6 weeks | Daily dose of 400 mg of chlorpromazine equivalents | Little or no symptomatic improvement as determined by clinical case notes | NS | NS |
| Istanbul (Turkey) | DSM-IV diagnosis of schizophrenia | 2 | At least two different antipsychotics | ≥6 weeks | At least the medium dosage of the therapeutic range | Persistent psychotic symptoms determined by a comparison of current and previous BPRS scores, alongside opinion of family member and treating psychiatrist | NS | NS |
| Lausanne (Switzerland) | Threshold criteria for psychosis as defined by the CAARMS scale | 2 | At least two different antipsychotics | ≥6 weeks | At least the mid-point of the licensed therapeutic range | A rating of at least moderate severity as rated by the PANSS (minimum of 4 on at least 2 positive-subscale items or a minimum of 6 on 1 positive-subscale item) for at least 12 weeks | At least moderate functional impairment measured using the GAF scale | Excluded those with poor compliance |
| TOP (Oslo, Norway) | DSM-IV diagnosis of schizophrenia, schizophreniform disorder, schizoaffective disorder, psychosis not otherwise specified (NOS), delusional disorder, brief psychosis or major affective disorder with mood incongruent psychotic symptoms | 2 | At least two different antipsychotics | ≥6 weeks | NS | A rating of at least moderate severity as rated by the PANSS (minimum of 4 on at least 2 positive-subscale items (or a minimum of 6 on 1 positive-subscale item) for at least 12 weeks | At least moderate functional impairment measured using the GAF scale | NS |
| Paris (France) | ICD-10 diagnosis of F20-F33 | 2 | At least two different antipsychotics | ≥6 weeks | At least the mid-point of the licensed therapeutic range | At least moderate severity, as rated by the PANSS, for at least 12 weeks | At least moderate functional impairment measured using the GAF scale | NS |
| Prague (Czech Republic) | ICD-10 diagnosis of F20 or F23 | 2 | At least two different antipsychotics | ≥6 weeks | At least the mid-point of the licensed therapeutic range | NS | NS | NS |
| Santander (Spain) | DSM-IV diagnosis of non-affective psychosis | 2 | At least two different antipsychotics | ≥6 weeks | At least the mid-point of the licensed therapeutic range | A rating of at least moderate severity, as rated by the PANSS, for at least 12 weeks | At least moderate functional impairment measured using the GAF scale | NS |
| Sao Paulo (Brazil)^2^ | DSM-IV diagnosis of psychosis | NS | NS | NS | NS | NS | NS | NS |
| West London (London, UK)^2^ | DSM-IV diagnosis of psychosis | NS | NS | NS | NS | NS | NS | NS |
| NB: ^1^ Excluded those who were intolerant of antipsychotic medications or those who self-discontinued medication; ^2^ Only clozapine prescription could be used to determine TR. | | | | | | | | |

| Supplementary Table 7. Average apparent performance measures for each cohort after repeated 5-fold validation (5 folds, 50 repeats). | | | |
| --- | --- | --- | --- |
| **Cohort** | **Alpha** | **Beta** | **AUC** |
| AESOP London | -0.1784 | 1.9323 | 0.6389 |
| Belfast | 0.6042 | 1.8443 | 0.6354 |
| Bologna | -0.6522 | -42.2914 | 0.6472 |
| GAP London | 0.1358 | 19.4901 | 0.7118 |
| Istanbul | 0.3861 | 1.2443 | 0.5916 |
| Lausanne | 0.4860 | 0.4225 | 0.5793 |
| Paris | -0.6921 | 2.5639 | 0.6193 |
| Prague | 0.3106 | 244.2620 | 0.7657 |
| Santander | 1.5056 | 5.1932 | 0.6558 |
| West London | -1.7783 | 3.3491 | 0.6602 |
| NB: Beta is the calibration slope and alpha is the calibration-in-the-large. | | | |

| Supplementary Table 8. Apparent and corrected performance measures for the 1SE logistic LASSO regression, when using both a 50% threshold and the ‘best’ threshold. | | | | |
| --- | --- | --- | --- | --- |
|  | **Apparent Performance** | | **Corrected Performance** | |
|  | **50% Threshold** | **Best (20.21%) Threshold** | **50% Threshold** | **Best (20.21%) Threshold** |
| Performance Measure |  |  |  |  |
| Alpha | 0.000003 | | 0.0372 | |
| Beta | 1.8171 | | 1.3469 | |
| AUC | 0.6529 | | 0.5889 | |
| Accuracy (%; 95%CI) | 0.8263  (0.8100, 0.8420) | 0.7130 (0.6940, 0.7320) | 0.7566 | 0.6433 |
| Sensitivity (%) | 0 | 0.4831 | 0 | 0.4831 |
| Specificity (%) | 1 | 0.7619 | 1 | 0.7619 |
| Positive Predictive Value (%) | NA | 0.2990 | NA | 0.2749 |
| Negative Predictive Value (%) | 0.8263 | 0.8752 | 0.8263 | 0.8755 |
| NB: Beta is the calibration slope and alpha is the calibration-in-the-large.  Abbreviations: AUC, area under the receiver operating characteristic curve. | | | | |

| Supplementary Table 9. Clozapine Model: Apparent and corrected performance measures for the 1SE logistic LASSO regression, when using both a 50% threshold and the ‘best’ threshold. | | | | |
| --- | --- | --- | --- | --- |
|  | **Apparent Performance** | | **Corrected Performance** | |
|  | **50% Threshold** | **Best (12.45%) Threshold** | **50% Threshold** | **Best (12.45%) Threshold** |
| Performance Measure |  |  |  |  |
| Alpha | 0.00004 | | 0.0132 | |
| Beta | 1.7317 | | 1.3968 | |
| AUC | 0.6463 | | 0.6109 | |
| Accuracy (%; 95%CI) | 0.88 (0.865, 0.894) | 0.568 (0.546, 0.59) | 0.8699 | 0.5579 |
| Sensitivity (%) | 0 | 0.6597 | 0 | 0.6597 |
| Specificity (%) | 1 | 0.5561 | 1 | 0.5561 |
| Positive Predictive Value (%) | NA | 0.1683 | NA | 0.1683 |
| Negative Predictive Value (%) | 0.8802 | 0.9231 | 0.8802 | 0.9241 |
| NB: Beta is the calibration slope and alpha is the calibration-in-the-large.  Abbreviations: AUC, area under the receiver operating characteristic curve. | | | | |

| Supplementary Table 10. Schizophrenia Model: Apparent and corrected performance measures for the 1SE logistic LASSO regression, when using both a 50% threshold and the ‘best’ threshold. | | | | |
| --- | --- | --- | --- | --- |
|  | **Apparent Performance** | | **Corrected Performance** | |
|  | **50% Threshold** | **Best (19.08%) Threshold** | **50% Threshold** | **Best (19.08%) Threshold** |
| Performance Measure |  |  |  |  |
| Alpha | 0.00007 | | 0.0041 | |
| Beta | 3.2146 | | 2.4569 | |
| AUC | 0.6378 | | 0.5459 | |
| Accuracy (%; 95%CI) | 0.81 (0.776, 0.841) | 0.552 (0.51, 0.593) | 0.7477 | 0.4897 |
| Sensitivity (%) | 0 | 0.7364 | 0.0148 | 0.7512 |
| Specificity (%) | 1 | 0.5085 | 0.9894 | 0.4980 |
| Positive Predictive Value (%) | NA | 0.2596 | NA | 0.2173 |
| Negative Predictive Value (%) | 0.8103 | 0.8918 | 0.8111 | 0.8713 |
| NB: Beta is the calibration slope and alpha is the calibration-in-the-large.  Abbreviations: AUC, area under the receiver operating characteristic curve. | | | | |


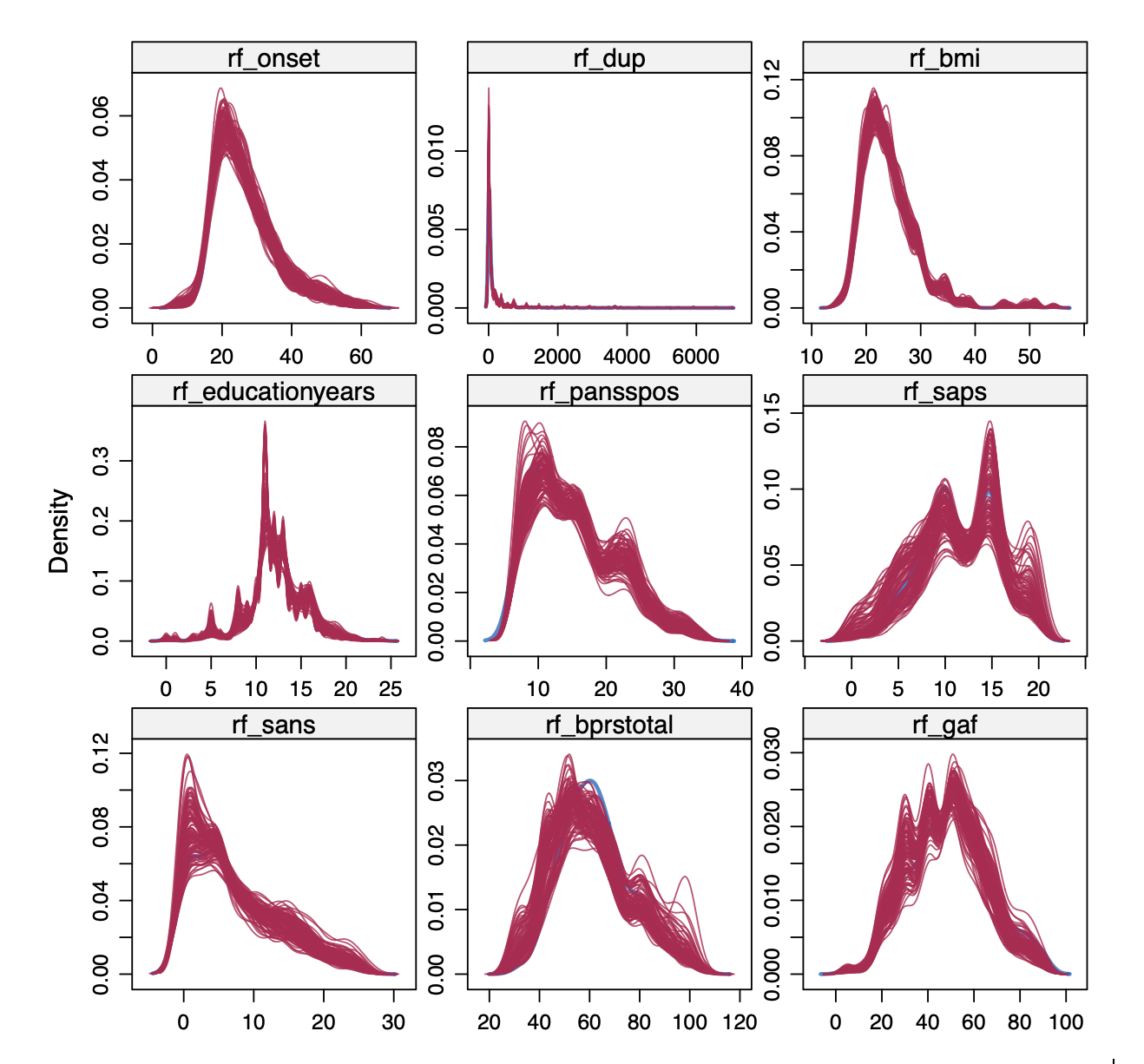


Figure 1. Multiple imputation using chained equations (MICE) density plots for imputed continuous variables. The density of the imputed data for each imputed dataset is showed in magenta while the density of the observed data is showed in blue.

Figure 2. Multiple imputation using chained equations (MICE) chain plots showing trace lines for the mean (left) and standard deviation (right) of the imputed values. The x-axis shows the number of iterations in the burn-in period.


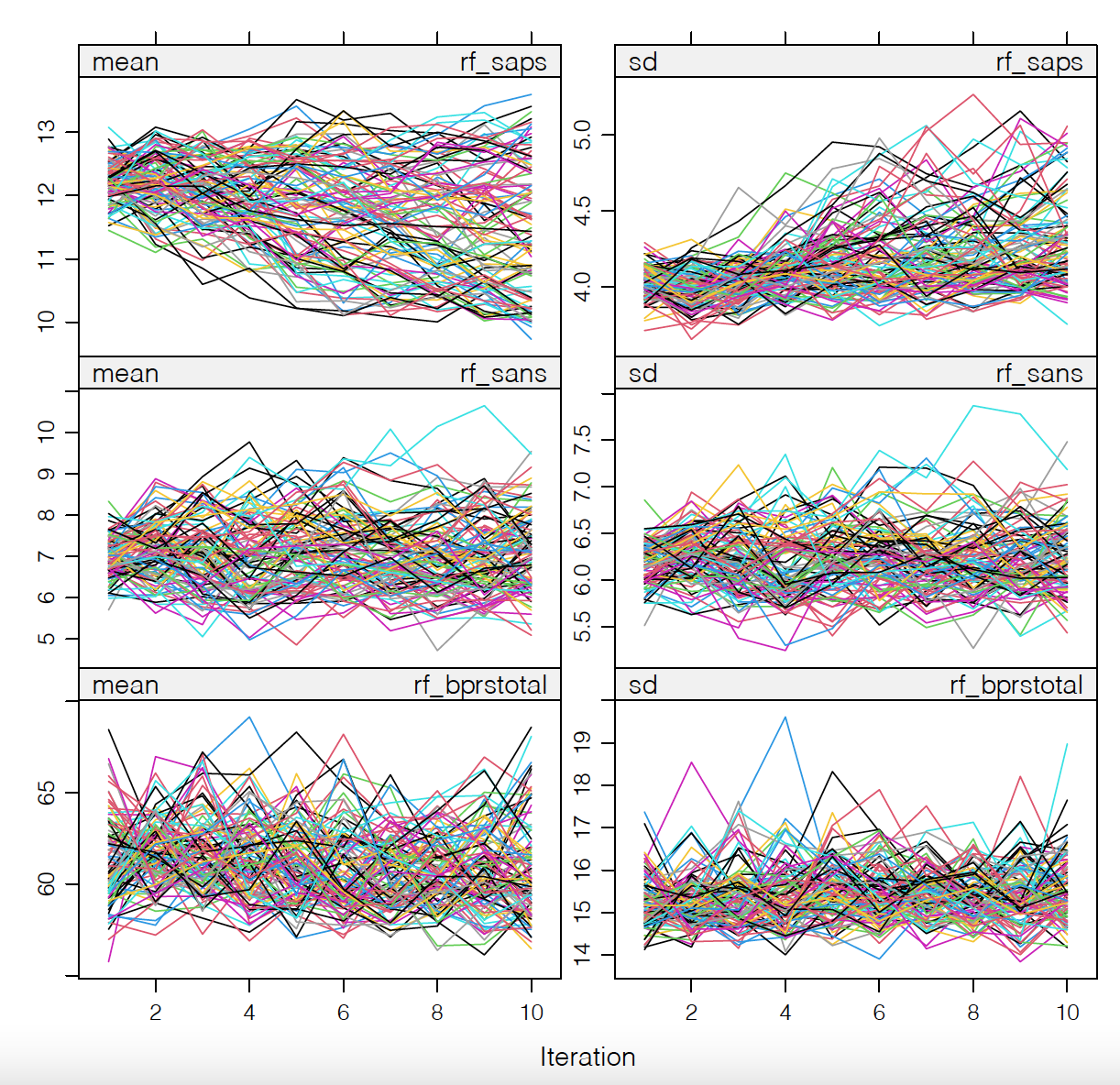

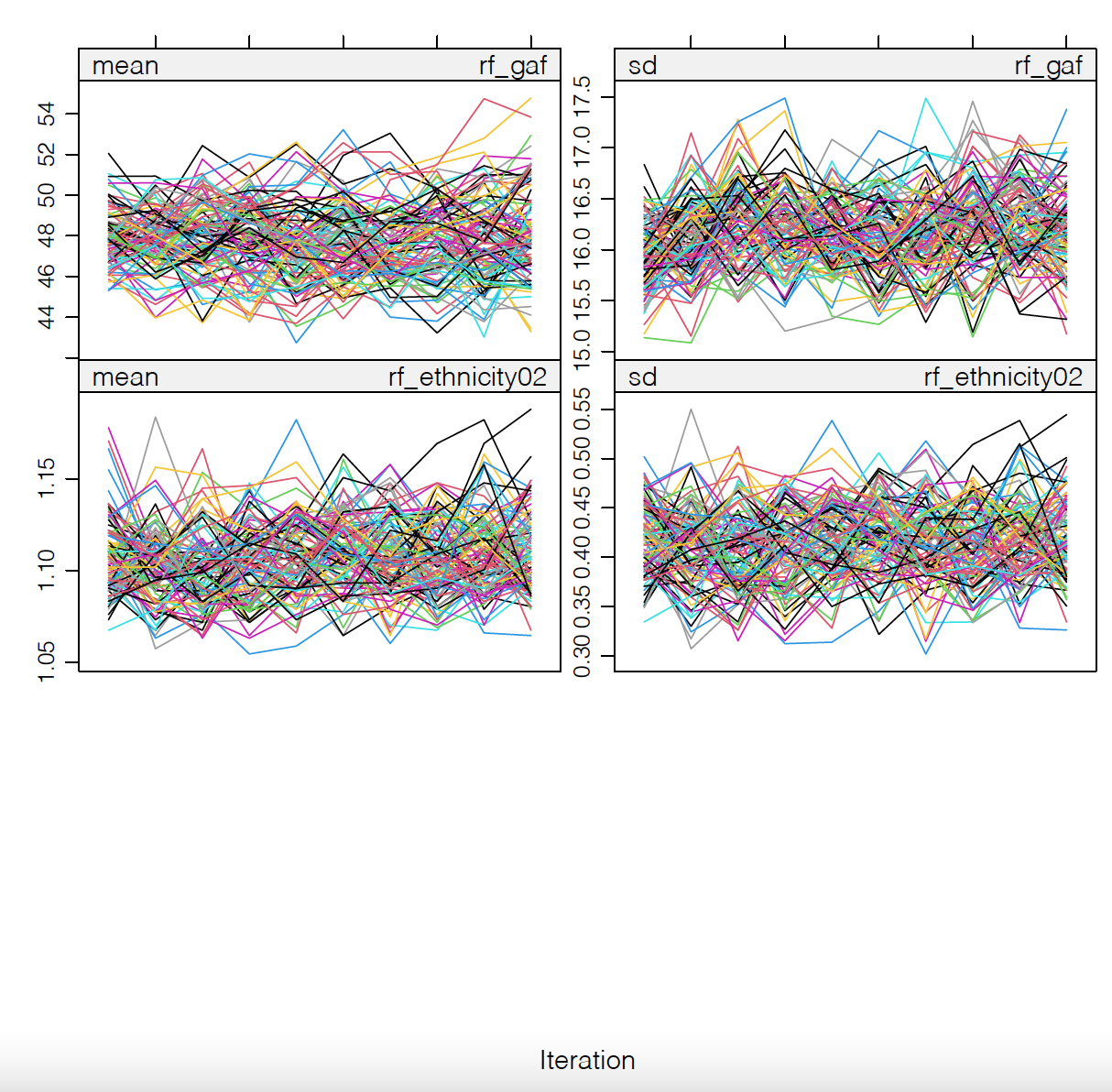

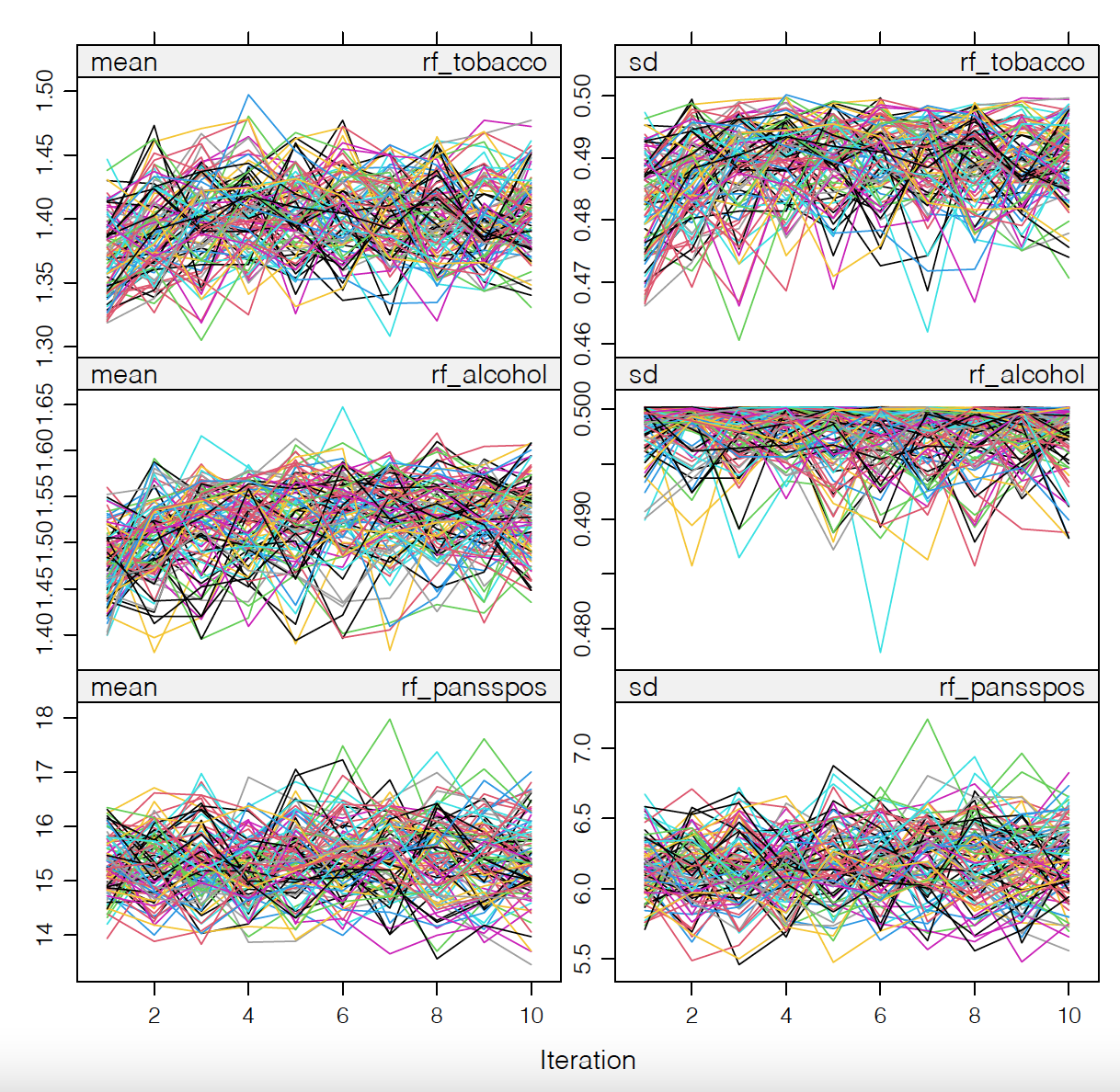

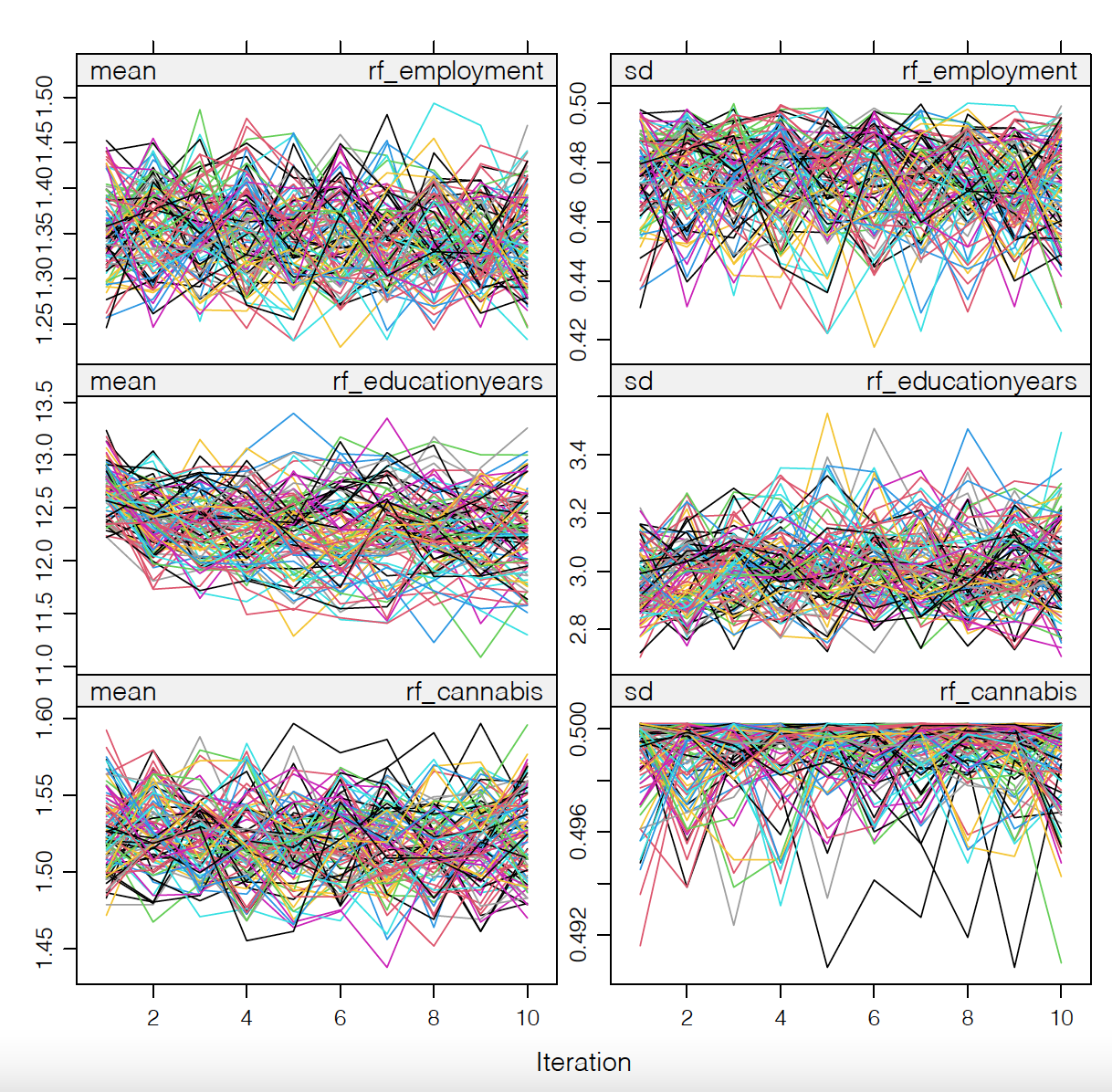

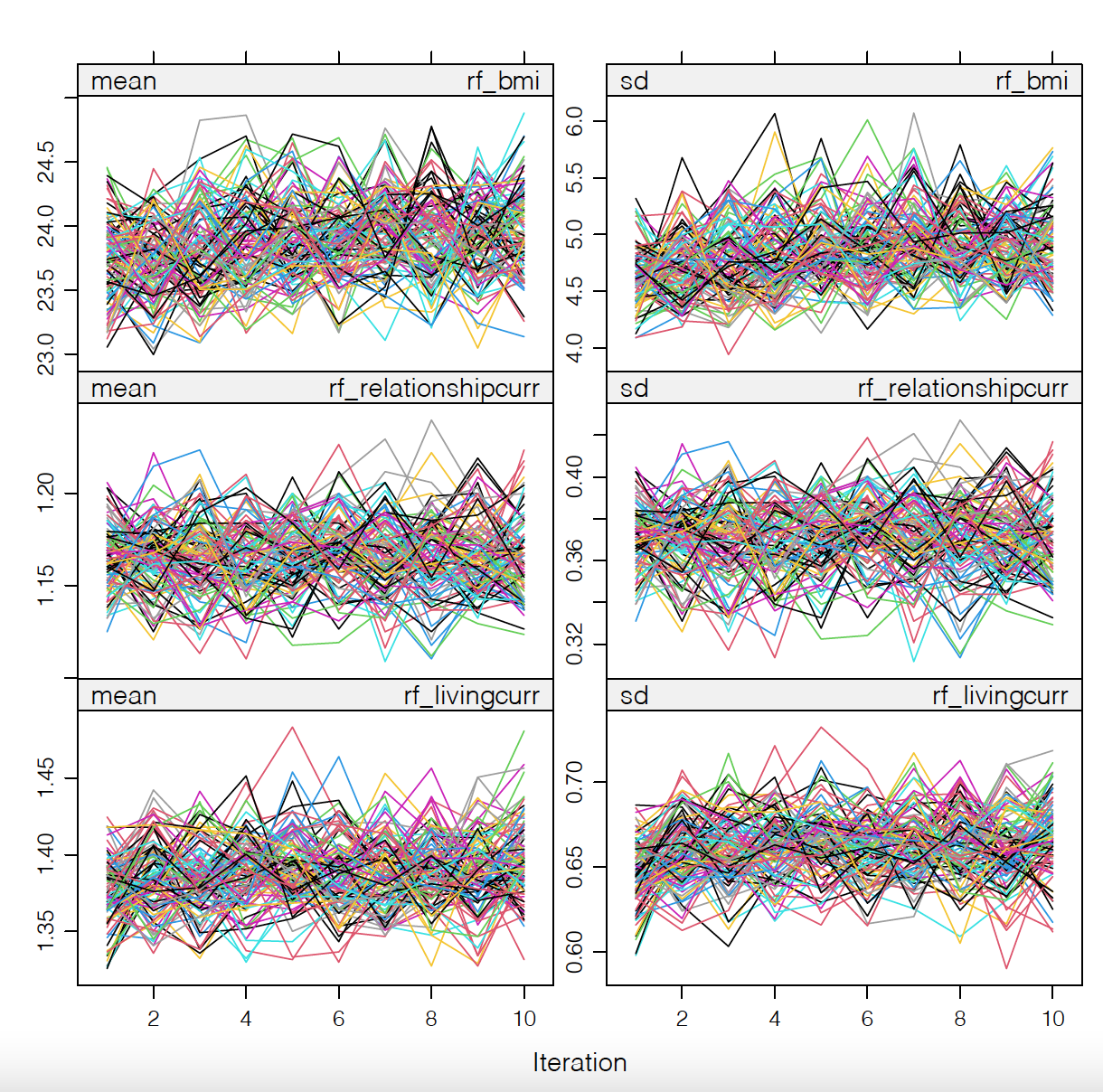

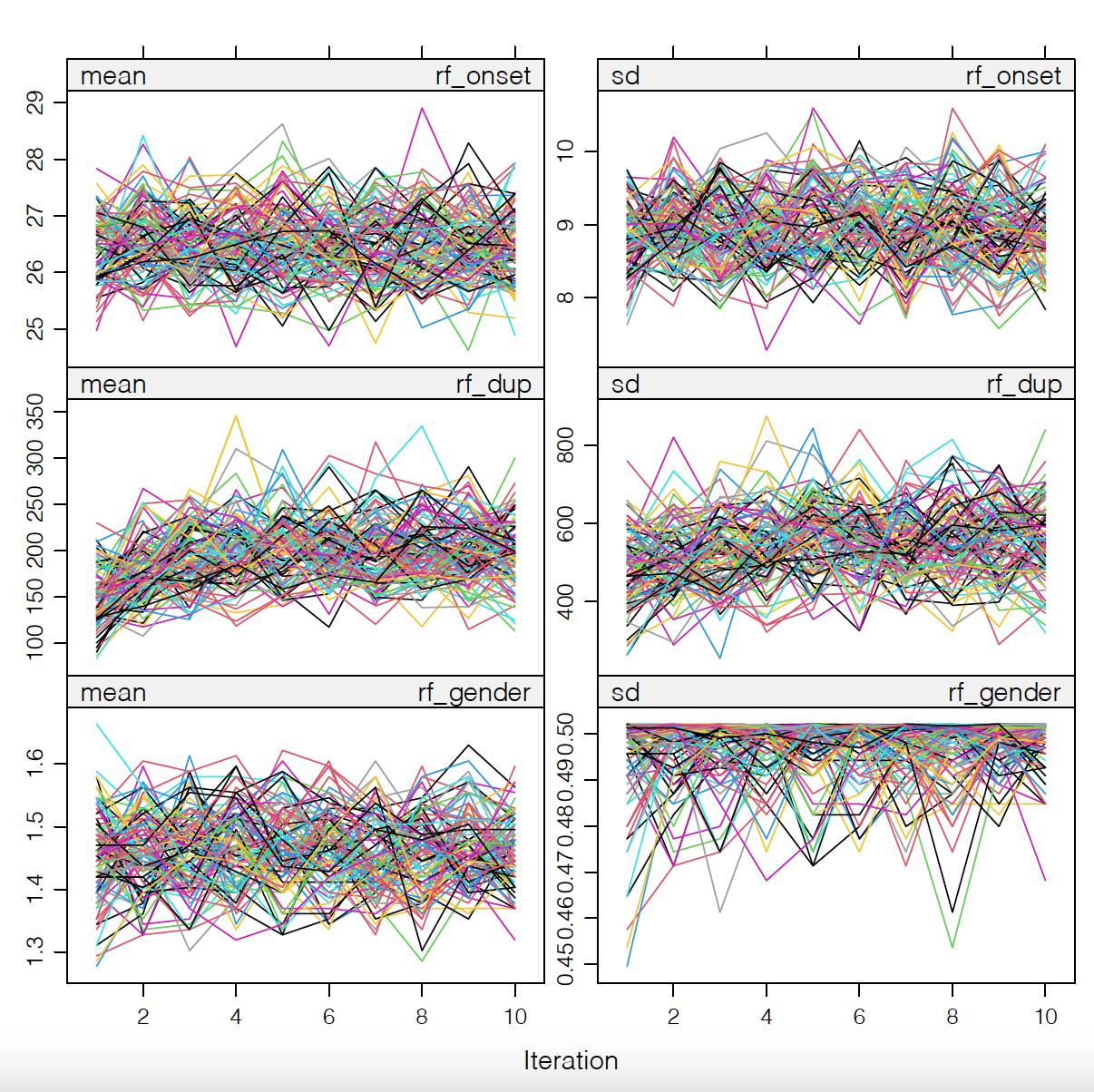

Figure 3. Plot showing lambda plotted against the receiver operating characteristic curve.

Figure 4. Plot showing the best threshold and apparent AUC for the 1SE logistic LASSO regression.
